# Satellite cell‐specific ablation of *Cdon* impairs integrin activation, FGF signalling, and muscle regeneration

**DOI:** 10.1002/jcsm.12563

**Published:** 2020-02-27

**Authors:** Ju‐Hyeon Bae, Mingi Hong, Hyeon‐Ju Jeong, Hyebeen Kim, Sang‐Jin Lee, Dongryeol Ryu, Gyu‐Un Bae, Sung Chun Cho, Young‐Sam Lee, Robert S. Krauss, Jong‐Sun Kang

**Affiliations:** ^1^ Department of Molecular Cell Biology Sungkyunkwan University School of Medicine Suwon Republic of Korea; ^2^ Single Cell Network Research Center Sungkyunkwan University School of Medicine Suwon Republic of Korea; ^3^ Department of Cell, Developmental, and Regenerative Biology Icahn School of Medicine at Mount Sinai New York NY USA; ^4^ Research Institute of Pharmaceutical Science, College of Pharmacy Sookmyung Women's University Seoul Republic of Korea; ^5^ Samsung Biomedical Research Institute Samsung Medical Center Seoul Republic of Korea; ^6^ Well Aging Research Center DGIST Daegu Republic of Korea; ^7^ Department of New Biology DGIST Daegu Republic of Korea

**Keywords:** Satellite cell, Muscle regeneration, Cdon, Cellular senescence, FGFR, Growth factor signalling

## Abstract

**Background:**

Perturbation in cell adhesion and growth factor signalling in satellite cells results in decreased muscle regenerative capacity. Cdon (also called Cdo) is a component of cell adhesion complexes implicated in myogenic differentiation, but its role in muscle regeneration remains to be determined.

**Methods:**

We generated inducible satellite cell‐specific Cdon ablation in mice by utilizing a conditional *Cdon* allele and *Pax7 ^CreERT2^*. To induce Cdon ablation, mice were intraperitoneally injected with tamoxifen (tmx). Using cardiotoxin‐induced muscle injury, the effect of Cdon depletion on satellite cell function was examined by histochemistry, immunostaining, and 5‐ethynyl‐2'‐deoxyuridine (EdU) incorporation assay. Isolated myofibers or myoblasts were utilized to determine stem cell function and senescence. To determine pathways related to Cdon deletion, injured muscles were subjected to RNA sequencing analysis.

**Results:**

Satellite cell‐specific *Cdon* ablation causes impaired muscle regeneration with fibrosis, likely attributable to decreased proliferation, and senescence, of satellite cells. Cultured Cdon‐depleted myofibers exhibited 32 ± 9.6% of EdU‐positive satellite cells compared with 58 ± 4.4% satellite cells in control myofibers (*P* < 0.05). About 32.5 ± 3.7% Cdon‐ablated myoblasts were positive for senescence‐associated β‐galactosidase (SA‐β‐gal) while only 3.6 ± 0.5% of control satellite cells were positive (*P* < 0.001). Transcriptome analysis of muscles at post‐injury Day 4 revealed alterations in genes related to mitogen‐activated protein kinase signalling (*P* < 8.29 e^−5^) and extracellular matrix (*P* < 2.65 e^−24^). Consistent with this, Cdon‐depleted tibialis anterior muscles had reduced phosphorylated extracellular signal‐regulated kinase (p‐ERK) protein levels and expression of ERK targets, such as *Fos* (0.23‐fold) and *Egr1* (0.31‐fold), relative to mock‐treated control muscles (*P* < 0.001). Cdon‐depleted myoblasts exhibited impaired ERK activation in response to basic fibroblast growth factor. Cdon ablation resulted in decreased and/or mislocalized integrin β1 activation in satellite cells (weak or mislocalized integrin1 in tmx = 38.7 ± 1.9%, mock = 21.5 ± 6%, *P* < 0.05), previously linked with reduced fibroblast growth factor (FGF) responsiveness in aged satellite cells. In mechanistic studies, Cdon interacted with and regulated cell surface localization of FGFR1 and FGFR4, likely contributing to FGF responsiveness of satellite cells. Satellite cells from a progeria model, *Zmpste24* ^−/−^ myofibers, showed decreased Cdon levels (Cdon‐positive cells in Zmpste24^−/−^ = 63.3 ± 11%, wild type = 90 ± 7.7%, *P* < 0.05) and integrin β1 activation (weak or mislocalized integrin β1 in Zmpste24^−/−^ = 64 ± 6.9%, wild type = 17.4 ± 5.9%, *P* < 0.01).

**Conclusions:**

Cdon deficiency in satellite cells causes impaired proliferation of satellite cells and muscle regeneration via aberrant integrin and FGFR signalling.

## Introduction

Skeletal muscle has a resilient regenerative capacity primarily governed by stem cells, called satellite cells. Normally, satellite cells are kept in a quiescent state and capable of self‐renewal and differentiation upon injury‐stimulated activation.^1^, ^2^ Quiescent satellite cells express Pax7 and when activated, express myogenic transcription factors, such as Myf5 and MyoD. This is followed by expansion and differentiation of progenitor cells, coinciding with decreased Pax7 and increased Myogenin expression.^3^ The ability of satellite cells to proliferate and return to quiescence is critical for the maintenance of the stem cell population.^4^, ^5^ Various muscle wasting conditions related to aging or dystrophy have been associated with a decline in the number and proliferative capacity of satellite cells.^5^, ^6^, ^7^ Satellite cells are remarkably resistant to cellular stress but progressively lose their ability to proliferate and differentiate with age. Satellite cell aging and dysfunction have been linked with cellular senescence driven by the accumulation of DNA damage and irreversible cell cycle arrest.^7^, ^8^, ^9^ Senescent cells have characteristic features such as flat appearance, senescence‐associated‐β‐galactosidase (SA‐β‐gal) activity, and upregulation of cyclin‐dependent kinase inhibitors p16^Ink4a^ (p16) and p21^Waf1^ (p21).^10^ In addition, senescent cells produce the senescence‐associated secretory phenotype (SASP), producing inflammatory cytokines and insulin‐like growth factor binding proteins. Although the dysregulation of cell cycle inhibitors appears to be the critical mechanism underlying cellular senescence,^7^, ^9^ the molecular mechanisms for satellite cell senescence are still largely unknown.

Several niche and cell‐intrinsic factors have been linked to satellite cell dysfunction.^7^, ^8^, ^11^, ^12^ Secreted factors like cytokines and growth factors, including fibroblast growth factor (FGF) family members, have been shown to play essential roles in satellite cell activation and proliferation upon injury.^13^, ^14^, ^15^ Accumulating evidence suggests that perturbed growth factor signalling is associated with satellite cell dysfunction, leading to muscle loss and weakness related to aging.^16^, ^17^, ^18^, ^19^ In addition, the interaction of satellite cells with their niche, through adhesion to myofibers via N‐cadherin and M‐cadherin or through adhesion to the basal lamina via integrins plays essential roles in satellite cell function.^20^, ^21^ Removal of N‐cadherin and M‐cadherin in satellite cells results in activation of quiescent satellite cells without injury, whereas deletion of integrin β1 or decreased fibronectin has been linked with satellite cell aging and decreased FGF response, resulting in impaired self‐renewal and proliferation capacity.^20^, ^22^ Furthermore, alteration in polarized localization of active integrin β1 (Itgb1*) is associated with impaired responsiveness of aged satellite cells to FGF, contributing to reduced regenerative capacity.^20^ However, the underlying regulatory mechanism of this polarized activation of integrin signalling is currently unclear.

The cell surface protein Cdon (also called Cdo) forms complexes with N‐cadherin at contact sites between skeletal myoblasts, and this interaction is critical for Cdon's promyogenic function.^23^ Consistent with its promyogenic role, *Cdon*‐null mice exhibit delayed skeletal muscle development, and Cdon‐deficient myoblasts differentiate less efficiently with decreased levels of muscle‐specific proteins.^24^, ^25^ In addition, the promyogenic function of Cdon appears to be dependent on integrin/focal adhesion kinase (FAK) signalling.^26^ Based on these observations, it is conceivable that Cdon plays an important role in adult muscle regeneration. In this study, we set out to elucidate the *in vivo* function of Cdon in adult satellite cells. Here, we report that inducible ablation of Cdon in satellite cells results in impaired muscle regeneration with fibrosis in a repetitive cardiotoxin (CTX)‐triggered injury model. Cdon deletion reduces the proliferative capacity of satellite cells. Furthermore, Cdon depletion in satellite cells or myoblasts elicit cellular senescence with enhanced expression of cell cycle inhibitors (p16 and p21), SASP, and phosphorylated‐γhistone2AX (pγH2AX), a marker for DNA double strand breaks. RNA sequencing analysis of muscles at post‐injury Day 4 revealed that Cdon ablation alters gene expression profiles related to the cellular response to cytokines and the MAPK signalling pathway without any significant change in the expression of myogenic regulators, such as Pax7, Myf5, MyoD, and Myogenin. Consistent with this, Cdon depletion elicits a decrease in FGF responsiveness and ERK activation, coinciding with reduction in the expression of immediate early genes. Interestingly, Cdon‐depleted satellite cells exhibit perturbed Itgb1* levels and localization. Furthermore, Cdon and Itgb1* levels were either decreased or mislocalized in satellite cells on myofibers isolated from Zmpste24^−/−^ mice, a model of progeria. Mechanistic analyses demonstrate that Cdon forms complexes with FGFR1 and FGFR4, and Cdon depletion reduces cell surface levels of FGFRs, likely contributing to reduced FGF responsiveness. Taken together, these data argue that Cdon regulates satellite cell proliferation via modulation of integrin activation and FGFR‐mediated signalling.

## Methods

### Animal experiments

To generate *Cdon* conditional knockout mice, three embryonic stem (ES) cells lines with conditional knockout potential for *Cdon* were obtained from the European Conditional Mouse Mutagenesis Program. Southern blotting analysis was performed by standard protocols to confirm the conditional *Cdon* allele (*Cdon* ^*tm3a*^ in Supporting Information, *Figure* S1) in two of the three ES cell lines (C03 and H02). Mouse lines were generated from these ES lines by the Mouse Genetics and Gene Targeting Core of Icahn School of Medicine at Mount Sinai. Both lines were similar, and line H02 was used for further analysis. The probes for the Southern blots were derived by PCR with the following probes: NsiI forward, AATGGACTGGCGTTGTGTACTGG; NsiI reverse, GTTTGTGTGTGCCTGCCCTTTC; NheI forward, ATGGAGATGCTTGTGGATGGG; NheI reverse, AAAGCCTCATTTCAGCCCAGC. See the legend to *Figure* S1 for further details.

*Cdon* ^*fl/fl*^ mice were crossed with *Pax7* ^*CreERT2*^ mice (obtained from the Jackson laboratory). The primers used for genotyping are listed in *Table* S1. Tamoxifen (tmx) (Sigma‐Aldrich, 1.5 mM) was administered intraperitoneally every 2 days for five times prior to muscle injury. To induce the skeletal muscle regeneration, mice were anaesthetized with a 1–2% isoflurane followed by CTX (Sigma‐Aldrich, 10 μM) injection into tibialis anterior (TA) muscle in a volume of 25 μl. *Zmpste24* ^−*/*−^ mice (kindly gifted by Dr. Zhongjun Zhou, University of Hong Kong) and wild type (WT) control mice were sacrificed to analyze satellite cells. All animal experiments were approved by the Institutional Animal Care and Research Advisory Committee at Sungkyunkwan University School of Medicine Laboratory Animal Research Center and complied with the regulations by the institutional Ethics Committee.

### Cell culture and single myofiber isolation

C2C12 myoblasts and 293 T cells were cultured as previously described.^27^ To determine the response to basic FGF (bFGF; Sigma‐Aldrich), C2C12 myoblasts were changed from Dulbecco's Modified Eagle Medium (DMEM) containing 15% foetal bovine serum (FBS) to DMEM containing 0.2% bovine serum albumin for overnight, followed by 5 ng/mL bFGF treatment as indicated. For adhesive culture, C2C12 cells were cultured either on matrigel (Corning) or poly‐L‐lysine (PLL) (Sigma‐Aldrich) coated petri dishes for 2 days. Primary myoblasts were isolated from hindlimbs of *Cdon* ^*+/+*^, *Cdon* ^*−/−*^, and *Cdon* ^*fl/fl*^ *;Pax7* ^*CreERT2*^ mice as previously described.^28^ For the Cre recombinase‐mediated *in vitro* deletion of Cdon, 4‐hydroxytamoxifen (4‐OHT; Sigma‐Aldrich, 1 µM) were treated for 4 days after amplification of isolated *Cdon* ^*fl/fl*^ *;Pax7* ^*CreERT2*^ primary myoblasts in F10 medium (Gibco) containing bFGF (2.5 ng/mL) and 20% FBS. Single myofiber isolation was performed as previously described.^27^ Briefly, extensor digitorum longus (EDL) muscles were digested with collagenase 2 (Worthington, 400 unit/mL), followed by seeding on matrigel‐coated plates in DMEM medium containing 20% FBS. Lentivirus‐expressing shRNA Cdon (shCdon) was generated with a modified lentiviral vector derived from pLKO.1 (Sigma‐Aldrich) in HEK 293 T cells using helper plasmids pCMV‐VSV‐G and pCMV dR 8.9. The shRNA sequences for Cdon used in this study are as previously reported.^28^

### Tissue preparation, histology, and immunostaining

For histology and immunostaining of muscles, snap freezing and cryosections were carried out as previously described.^29^, ^30^ To assess the structure and fibrosis, fixed cryosections were stained with Mayer's haematoxylin and eosin (BBC Biomedical) and Picro‐Sirius red (Abcam) according to the manufacturer's protocol. Immunostaining was performed as previously described.^29^, ^30^ Cultured cells, single myofibers, and frozen muscle sections were fixed, permeabilized and processed for incubation with primary antibodies against Cdon, Pax7, Laminin, eMyHC, activated‐integrin β1 (*Itgb1), cleaved Caspase3, pγH2AX, and secondary antibodies (Thermo Fisher). SA‐β‐gal staining was conducted by using SA‐β‐gal kit (Cell Signaling Technology) as previously described.^31^ SA‐β‐gal staining for muscle tissue was performed as previously described.^32^ Briefly, freshly frozen sections were fixed, followed by incubating X‐gal mixture (pH 5.5) for 2 days at 37°C. 5‐Ethynyl‐2'‐deoxyuridine, a nucleoside analog of thymidine (EdU) incorporation assay was performed by adding EdU with a final concentration of 10 μM for 2 h before fixation and then analyzed using the Click‐iT EdU kit (Invitrogen) according to the manufacturer's protocol. Images were acquired under LSM‐710 Meta confocal microscope or Nikon ECLIPSE TE2000‐U microscope.

### Real‐time polymerase chain reaction analysis and RNA sequencing

Quantitative real‐time PCR analysis was examined as previously described.^33^ cDNA was obtained from total RNA using a PrimeScript RT reagent kit (TAKARA) and analyzed by quantitative real‐time PCR using SYBR Premix Ex Tag^TM^ (TAKARA). The primers sequences are listed in *Table* S1, and each transcript levels were normalized with ribosomal protein L32. RNA sequencing analysis was performed with the Illumina NextSeq500 (ebiogen, Korea). All gene set enrichment analysis (GSEA) plots including GSEA enrichment plot and map were generated with the GSEA software (Broad Institute; software.broadinstitute.org/gsea/).

### Protein analysis

Immunoblot and immunoprecipitation analyses were carried out as previously described.^30^ Briefly, tissues and cells were lysed in radioimmunoprecipitation assay (Thermo) buffer with complete protease inhibitor cocktail (Roche) and separated by sodium dodecyl sulfate–polyacrylamide gel electrophoresis. For immunoprecipitation, precleared cell extracts were incubated with primary antibodies overnight at 4°C, followed by incubation with protein A‐agarose or G‐agarose beads (Roche Diagnostics) for 1 h and washing three times with cell extraction buffer. Precipitates were analyzed by western blotting. Surface biotinylation was performed as previously explained.^34^ Briefly, biotinylated surface proteins were precipitated with streptavidine‐agarose beads (Pierce) and analyzed with sodium dodecyl sulfate–polyacrylamide gel electrophoresis. Primary antibodies are listed in *Table* S2.

### Statistical analysis

All values were analyzed using GraphPad Prism 7 or Excel spreadsheet. To assess the statistical difference, Student's *t*‐test with two‐tailed paired comparison was performed. In case of comparison groups as indicated in the figure legend, we determined the statistical significance by one‐way analysis of variance with Tukey post hoc test. Statistical significance was indicated as *P* < 0.05 (*), *P* < 0.01 (**), and *P* < 0.001(***). Error bar were represented by mean ± standard deviation (mean ± SD).

## Results

### Cdon ablation in satellite cells impairs muscle regeneration

**Figure 1:**
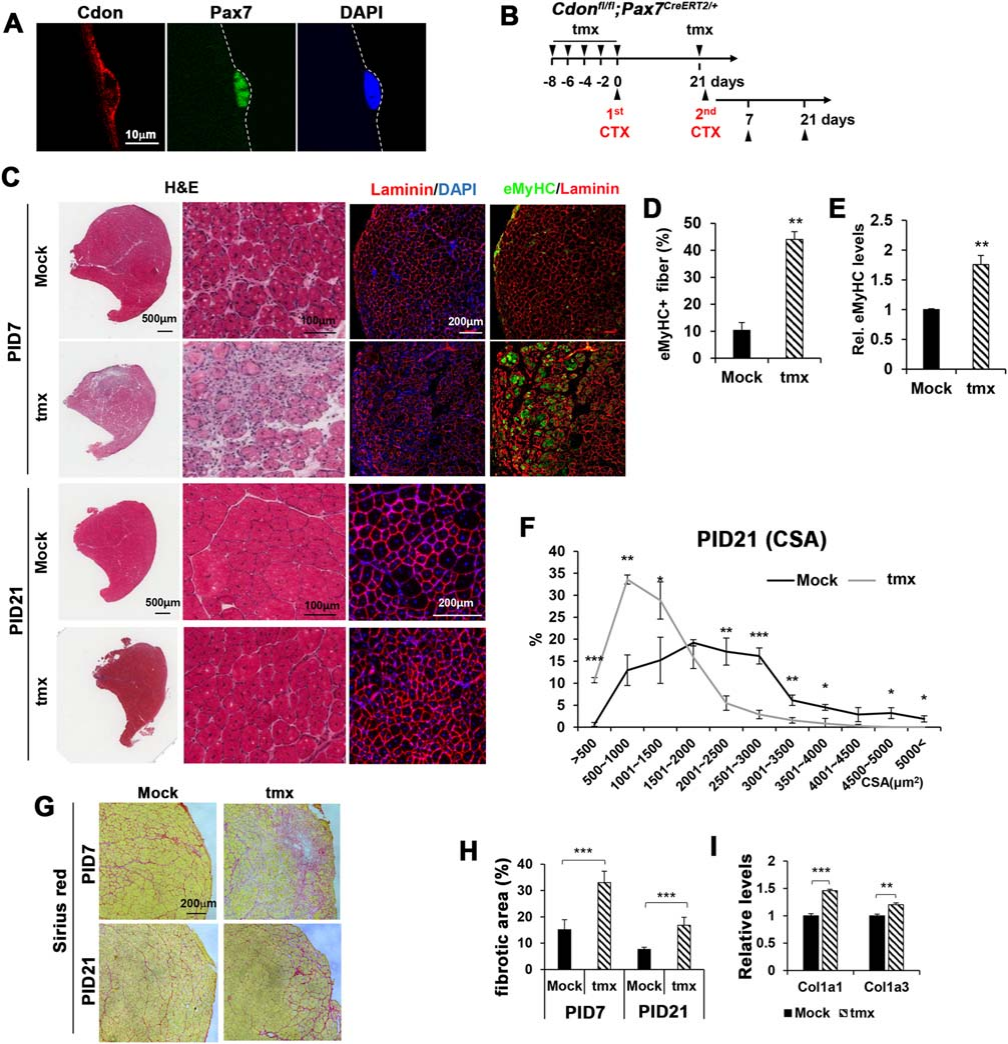
The ablation of Cdon in satellite cells impairs muscle regeneration. (A) Cdon (red) and Pax7 (green) immunostaining of satellite cell on single myofiber isolated from extensor digitorum longus (EDL) muscles. DAPI labels nucleus (blue). (B) The experimental scheme for repetitive muscle injury. *Cdon* ^*fl/fl*^ *;Pax7* ^*CreERT2*^ mice were injected intraperitoneally with tamoxifen (tmx) five times prior to cardiotoxin injection in the tibialis anterior (TA) muscle. (C) Histological analysis (haematoxylin and eosin, H&E) and immunostaining for laminin and embryonic myosin heavy chain (eMyHC) with mock or tmx‐treated TA muscles from 7 (PID7) or 21 days post the second injury. (D) Quantification of eMyHC‐positive myofibers in the injured area of PID7 muscles (*n* = 3, **P < 0.01). (E) Quantitative RT‐PCR for eMyHC with control and tmx‐treated muscles at PID7. L32 served as an internal control. The value of control muscles was set to 1. (*n* = 3, **P < 0.01). (F) Quantification of myofiber cross‐sectional area (CSA) of PID21 muscles. (*n* = 3, *P < 0.05, **P < 0.01, ***P < 0.001). (G) Sirius red staining to assess fibrosis. (H) The relative fibrotic area was assessed by using Image J software. (*n* = 4, ***P < 0.001, Student's *t*‐test). (I) Quantitative RT‐PCR for Col1a1 and Col1a3 with control and tmx‐treated muscles at PID7. The value of control muscles is set to 1. (*n* = 3, **P < 0.01, ***P < 0.001).

To investigate the role of Cdon in muscle regeneration, we have analyzed the expression of Cdon in satellite cells by immunostaining of myofibers isolated from EDL muscles. Cdon is expressed in Pax7‐positive satellite cells (*Figure* 1A), preferentially at the basal membrane (*Figure* S2). To define the *in vivo* function of Cdon in muscle regeneration, we generated mice carrying a floxed allele of *Cdon* (*Figure* S1). These mice were crossed with *Pax7* ^*CreERT2*^ mice, which express tmx‐inducible Cre recombinase in satellite cells but not other cells in skeletal muscle tissue^35^; 3‐month‐old *Cdon* ^*fl/fl*^ *;Pax7* ^*CreERT2*^ mice were injected five times with saline (mock) or tmx starting 8 days prior to the first CTX injury of TA muscles; 21 days after the first injury, a second injury was initiated (*Figure* 1B). Immunoblotting of TA muscles revealed that Cdon protein was depleted in tmx‐treated muscles 7 days after the second injury, compared with the mock saline‐treated muscles (*Figure* S3A and S3B). In addition, quantitative RT‐PCR analysis of isolated myoblasts and fibroblasts treated with mock or 4‐hydroxytamoxifen (4‐OHT) showed that Cdon transcription was specifically decreased in myoblasts (*Figure* S3C). Cdon ablation in satellite cells mildly impaired muscle regeneration as assessed by myofiber cross‐sectional area 21 days after the first injury (*Figure* S4A and S4B). After the second injury, a more severe impairment in muscle regeneration was observed (*Figure* 1C). Control muscles displayed myofibers with centrally localized nuclei, characteristic of regenerating muscles 7 days after the second CTX injury (PID7), while Cdon‐depleted muscles accumulated single cells in interstitial areas between regenerating myofibers. In addition, immunostaining for laminin and embryonic myosin heavy chain (eMyHC, a marker for immature myofibers) demonstrated that more myofibers in Cdon‐depleted muscles at PID7 were positive for eMyHC, compared with control muscles (*Figure* 1C and 1D). Consistent with this, eMyHC transcript levels were significantly increased in Cdon‐depleted muscles, relative to control muscles (*Figure* 1E). The myofiber size in Cdon‐depleted muscles 21 days after the second injury (PID21) was greatly reduced, compared with the control muscles (*Figure* 1C and 1F). Impaired muscle regeneration processes are frequently linked with aberrant extracellular matrix (ECM) remodelling and fibrosis.^36^, ^37^ In agreement with this notion, Sirius red staining showed that Cdon depletion in satellite cells led to an exacerbated fibrotic response at PID7 and PID21 (*Figure* 1G and 1H). Similarly, transcript levels of Collagen 1a1 (Col1a1) and Col1a3 were mildly but significantly increased in Cdon‐depleted muscles (*Figure* 1I). The weights of Cdon‐depleted muscles at PID7 or PID21 were also significantly decreased, compared with control muscles (*Figure* S5). Taken together, these data demonstrate that Cdon ablation impairs satellite cell function.

### Cdon ablation reduces proliferative and self‐renewal capacity of satellite cells

**Figure 2:**
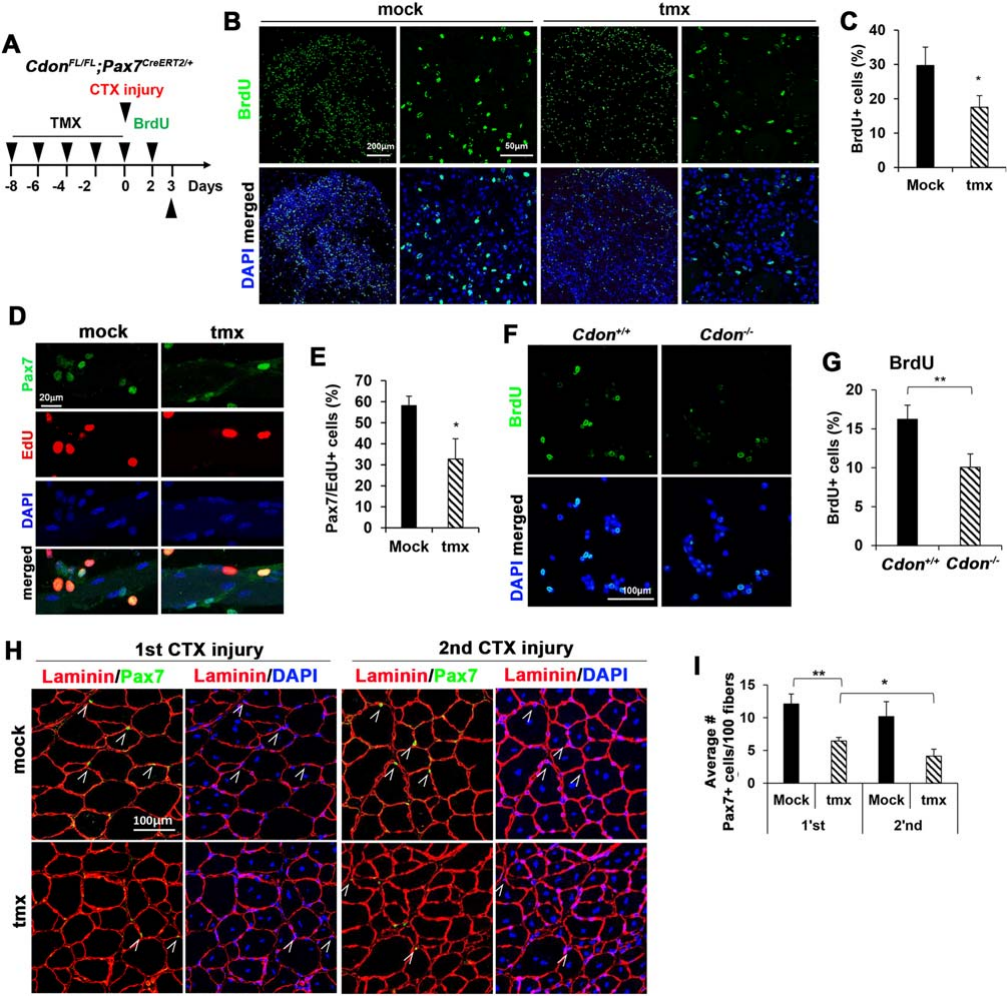
Loss of Cdon decreases the proliferation of satellite cells. (A) The experimental scheme. *Cdon* ^*fl/fl*^ *;Pax7* ^*CreERT2*^ mice were injected IP with tamoxifen five times followed by CTX injury and at PID2 TA muscles were subjected to BrdU incorporation for 1 day. (B, C) Immunostaining of TA muscles for BrdU incorporation (green). Nuclei were visualized by DAPI staining (blue). Quantification of BrdU‐positive cells and the values presented as percentile relative to total nuclei; ~1700 nuclei per sample were counted (*n* = 4, *P < 0.05). (D, E) Immunostaining for EdU (red) and Pax7 (green) of isolated EDL myofibers after 48 h of isolation. (*n* = 3, each group; ≥15 myofibers per animal, *P < 0.05). (F, G) Immunostaining for BrdU in *Cdon* ^*+/+*^ and *Cdon* ^*−/−*^ primary myoblasts labelled with BrdU for 20 min. (*n* = 5, **P < 0.01). (H) Immunostaining for laminin and Pax7 of muscle at PID21 after first injury and 2nd injury. The white arrowhead indicates Pax7‐positive cells. (I) Quantification of Pax7‐positive cells per 100 centrally nucleated myofibers (*n* = 3, three sections per animal, *P < 0.05, **P < 0.01).

To investigate the detailed mechanism underlying the impaired regenerative capacity of Cdon‐ablated satellite cells, we first analyzed their proliferative capacity. To do so, TA muscles of mock or tmx‐treated mice were injured and injected with bromodeoxyuridine (BrdU) 2 days after a single injury to label proliferating cells, followed by immunostaining analysis 24 h later (*Figure* 2A). Forty per cent of cells in control regenerating muscles were BrdU‐positive, while ~20% of cells in the tmx‐treated muscles were BrdU‐positive (*Figure* 2B and 2C). In addition, TA muscles of mock or tmx‐treated mice at 4 days after CTX injury were immunostained for Pax7 and Ki67 (*Figure* S6). In the control muscles, about 27% of cells were double positive for Pax7 and Ki67, while ~13% of cells in tmx‐treated muscles were double positive. To further examine this, single myofibers from EDL muscles were isolated from mock or tmx‐treated mice and cultured for 48 h in the presence of bFGF followed by 5‐ethynyl‐2'‐deoxyuridine (EdU) incorporation assay (*Figure* 2D and 2E). In agreement with the results from *in vivo* BrdU incorporation, EdU incorporation was significantly lower in satellite cells on myofibers from tmx‐treated mice, compared with control myofibers. Furthermore, *Cdon* ^*−/−*^ primary myoblasts exhibited significantly reduced BrdU incorporation relative to that of *Cdon* ^*+/+*^ myoblasts (*Figure* 2F and 2G). However, Cdon‐deficient myoblasts showed no overt signs of cell death, as measured by cleaved Caspase 3 immunostaining (*Figure* S7). Self‐renewal of satellite cells is critical for muscle regeneration and maintenance.^7^, ^9^ Mock or tmx‐treated TA muscle sections from PID21 were therefore immunostained for Pax7 and laminin, and the number of Pax7‐positive cells was quantified (*Figure* 2H and 2I). In control muscle sections, there were about 12 Pax7‐positive cells per 100 regenerating myofibers 21 days after the first injury, which decreased to ~10 such cells after the second injury. Cdon‐depleted muscles had a significantly decreased number, 5–6 Pax7‐positive cells per 100 regenerating myofibers 21 days after each injury. Taken together, these data reveal that Cdon depletion causes reduced proliferative and self‐renewal capacity of satellite cells.

### Cdon depletion in satellite cells elicits cellular senescence

**Figure 3:**
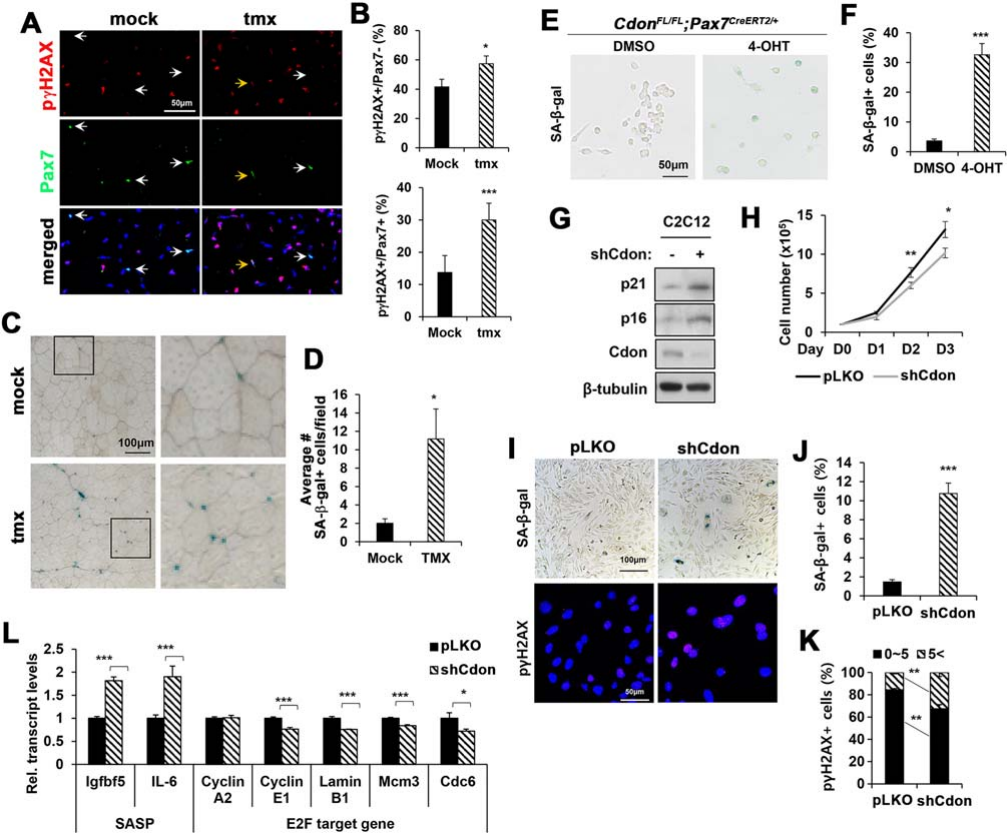
Cdon ablation in satellite cells induces cellular senescence of myoblasts. (A) Immunostaining of phospho‐γH2AX (pγH2AX) and Pax7 on muscle sections at PID21 after the second injury. (B) Quantification of pγH2AX‐positive cells in the Pax7‐negative or Pax7‐positive populations. (*n* = 4; mock, 170–276 nuclei per sample; tmx, 278–340 nuclei per sample, *P < 0.05, ***P < 0.001). (C, D) SA‐β‐gal staining of TA muscles at PID21 after the second injury (147–270 myofibers per sample were counted. *n* = 3, *P < 0.05). (E, F) SA‐β‐gal staining of isolated primary myoblasts from *Cdon* ^*FL/FL*^;*Pax7Cre* ^*ERT2/+*^ mice. Isolated cells were treated with 1 μM 4‐hydroxytamoxifen (4‐OHT) for 4 days. (*n* = 3, ***P < 0.001). (G) Immunoblot analysis for cell cycle inhibitors p21 and p16 in control or shCdon‐expressing lentivirus infected C2C12 myoblasts. (H) Growth curves of control and Cdon‐depleted C2C12 myoblasts. (*n* = 3, *P < 0.05, **P < 0.01). (I) SA‐β‐gal staining and pγH2AX immunostaining in control lentivirus‐expressing and shCdon‐expressing lentivirus‐infected C2C12 myoblasts. (J) Quantification for SA‐β‐gal‐positive cells in Panel I (*n* = 3, ***P < 0.001). (K) Quantification for pγH2AX foci of C2C12 cells in Panel I (*n* = 3, **P < 0.01). (L) Quantitative RT‐PCR for SASP and E2F target genes in control and Cdon‐depleted C2C12 myoblasts (*n* = 3, *P < 0.05, ***P < 0.001).

We next analyzed muscles at PID21 for production of Pax7 and pγH2AX (*Figure* 3A and 3B). Cells double positive for Pax7 and pγH2AX or positive for pγH2AX only were quantified. Cdon depletion resulted in an increased proportion of pγH2AX‐positive cells in both Pax7‐positive and Pax7‐negative populations, compared with controls. To address whether impaired self‐renewal and proliferative capacity in Cdon‐depleted satellite cells are associated with cellular senescence, mock and tmx‐treated muscle sections from PID21 were subjected to SA‐β‐gal staining (*Figure* 3C and 3D). SA‐β‐gal‐positive cells were rarely found in the control muscles, while Cdon‐depleted muscles averaged 11 SA‐β‐gal‐positive cells per field. To confirm the effect of Cdon depletion on cellular senescence, primary myoblasts isolated from *Cdon* ^*fl/fl*^ *;Pax7* ^*CreERT2*^ mice were treated *in vitro* with dimethyl sulfoxide or 4‐OHT for 4 days in the presence of bFGF and subjected to SA‐β‐gal staining (*Figure* 3E and 3F). More than 30% of Cdon‐depleted myoblasts were positive for SA‐β‐gal staining, while <5% of dimethyl sulfoxide‐treated control myoblasts were SA‐β‐gal positive. Additionally, *Cdon* ^*−/−*^ primary myoblasts exhibited significantly increased pγH2AX‐positive cells relative to that of *Cdon* ^*+/+*^ myoblasts (*Figure* S8). These data indicate that Cdon depletion resulted in cellular senescence and suggested it produces defective responsiveness to bFGF.

Similar to Cdon‐deficient satellite cells, C2C12 cells infected with a Cdon shRNA lentivirus expressed higher levels of p21 and p16 proteins, compared with control lentivirus‐infected cells (*Figure* 3G). Cdon‐depleted C2C12 cells also proliferated less efficiently than control cells (*Figure* 3H). SA‐β‐gal staining and pγH2AX immunostaining revealed that Cdon depletion significantly elevated SA‐β‐gal‐positive cells and pγH2AX‐foci, compared with control (*Figure* 3I–3K). To further examine this, qRT‐PCR analysis for SASP genes (Igfbp5 and interleukin‐6) and E2F target genes was performed in control or Cdon‐depleted C2C12 cells (*Figure* 3L). Similar to the SA‐β‐gal staining, the expression of Igfbp5 and interleukin‐6 was significantly increased in Cdon‐depleted cells. In contrast, the E2F target genes Cyclin E1, Lamin B1, Mcm3, and Cdc6 were mildly but significantly decreased in Cdon‐depleted cells, compared with the control cells. Taken together, these data suggest that Cdon depletion in satellite cells and myoblasts results in cellular senescence and impaired stem cell function, likely contributing to decreased muscle regeneration.

### Cdon depletion in satellite cells alters global gene expression profiles induced by muscle injury

**Figure 4:**
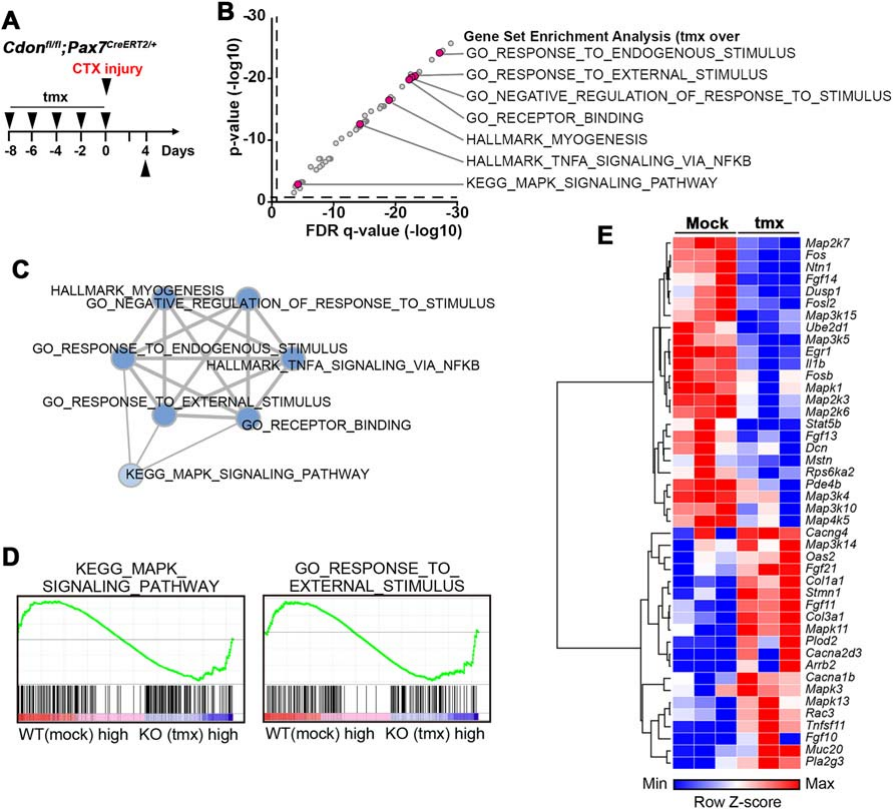
Alterations in global gene expression in Cdon‐depleted muscles at 4 days after CTX injury. (A) The experimental scheme for RNA sequencing of TA muscles at PID4. (B) Key gene sets showing statistical significance were displayed based on their nominal *P* value (‐log_10_) and FDR *q* value (‐log_10_). (C) Enrichment Map, a network‐based analysis, visualizing the association among key seven gene sets. (D–E) The presentative results of GSEA are displayed as enrichment plot and heat map showing upregulated or downregulated genes.

To determine the regulatory pathways underlying regeneration defects caused by Cdon deficiency, we performed RNA sequencing analysis with muscles of either mock‐injected or tmx‐injected mice 4 days after a single injury (*Figure* 4A). To identify groups of genes associated with observed phenotypes in Cdon‐deficient satellite cells, GSEA, as an unbiased approach, was performed (*Figure* 4B–4E). GSEA analysis revealed that gene sets implicated in endogenous stimulus, external stimulus, and negative regulation of response to stimulus were significantly altered (dashed lines indicate nominal *P* = 0.05 and false discovery rate *q* = 0.05) (*Figure* 4B and *Figure* S9). Moreover, KEGG pathway mapping showed that genes associated with arginine and proline metabolism, ECM‐receptor interaction, pathways in cancer, and MAPK signalling pathway were altered in Cdon‐depleted muscles. Enrichment Map (*Figure* 4C), a network‐based method for gene set enrichment visualization, revealed that the key seven gene sets are tightly associated with each other, implying that genes of those seven pathways underpin the phenotypes of Cdon‐deficient satellite cells. Additionally, the gene expression patterns of those gene sets (e.g. KEGG MAPK signalling pathway and GO Response to external stimulus) seemed to be divided in half (*Figure* 4D and 4E). Furthermore, genes involved in inflammatory response, ECM, negative regulation of cell population proliferation were perturbed in Cdon‐deleted muscles (*Figure* S10). Interestingly, the expression of myogenic regulatory factors, including *Myf5*, *Myogenin*, *Pax7*, *Pax3*, *Myod1*, and *Mef2c* were not significantly altered between control and Cdon‐deleted muscles (*Figure* S11A), suggesting satellite cell activation initiated normally.

### Cdon ablation in satellite cells impairs extracellular signal‐regulated kinase activation and fibroblast growth factor response

**Figure 5:**
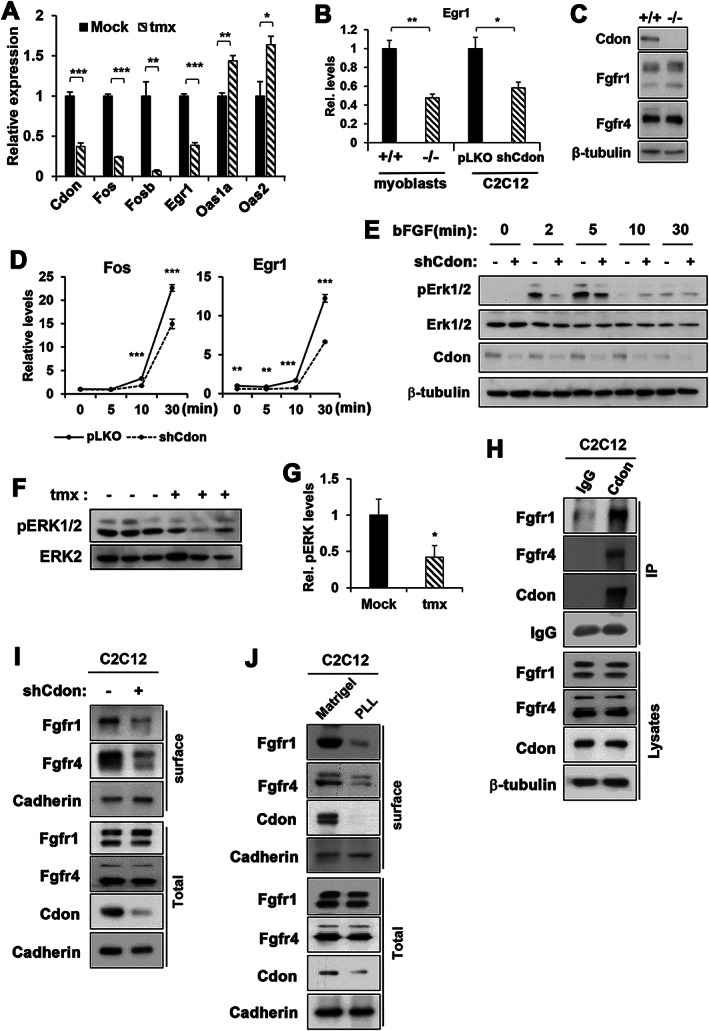
Cdon depletion causes reduced responsiveness to bFGF treatment and Cdon regulates FGFR1/4 surface expression. (A) qRT‐PCR analysis for the expression of ERK target genes (*Fos*, *Fosb*, *Egr1*, *Oas1a*, and s*Oas2*) and *Cdon* in Cdon‐depleted muscles. (*n* = 3, *p < 0.05, **P < 0.01, ***P < 0.001). (B) qRT‐PCR analysis for *Egr1* expression in primary myoblasts with indicated genotypes and control or Cdon‐depleted C2C12 cells. (*n* = 3, *p < 0.05, **P < 0.01). (C) Immunoblot analysis for FGFR1 and FGFR4 in primary myoblasts from *Cdon* ^*−/−*^ or *Cdon* ^*+/+*^ mice. β‐tubulin served as loading control. (D) qRT‐PCR analysis for *Fos* and *Egr1* in C2C12 myoblasts. Cells were serum starved overnight followed by 5 ng/mL bFGF treatment as indicated (*n* = 3, **P < 0.01, ***P < 0.001). (E) Immunoblot analysis for ERK activation in control and Cdon‐depleted C2C12 cells in response to bFGF treatment. (F, G) Immunoblot analysis for ERK and p‐ERK1/2 in TA muscles at PID4. The relative signal intensity of p‐ERK was plotted, and the value from the mock control was set to 1 (*n* = 3, *p < 0.05). (H) Coimmunoprecipitation of endogenous Cdon with FGFR1 and FGFR4 in C2C12 cells. (I) Control and Cdon‐depleted C2C12 cells were subjected to biotinylation and pull down with streptoavidin bead followed by immunoblotting for FGFR1, FGFR4, and cadherin as control. (J) C2C12 cells cultured on normal plastic and poly‐L‐lysine‐coated plates were subjected to biotinylation analysis and pull down with streptavidin bead followed by immunoblotting for Cdon, FGFR1, FGFR4, and cadherin.

To further examine whether decreased proliferation of Cdon‐depleted satellite cells is because of perturbed MAPK signalling, qRT‐PCR analysis of genes regulated by the ERK was performed. Expression of *Fosb*, *Fos*, and *Egr1*, immediate early response genes induced by ERK, was significantly decreased, while expression of *Oas1a* and *Oas2*, which is inhibited by ERK,^38^ was elevated (*Figure* 5A). Furthermore, Cdon‐deficient primary myoblasts cultured in the presence of bFGF and Cdon‐depleted C2C12 cells in growth medium exhibited significantly decreased Egr1 expression (*Figure* 5B). The RNA sequencing data showed no significant alteration in expression of growth factors (various FGFs and HGF) or FGF receptors that are related to ERK activation (*Figure* S11B). Similarly, *Cdon* ^*+/+*^ and *Cdon* ^*−/−*^ myoblasts had similar levels of FGFR1 and FGFR4 proteins (*Figure* 5C). To explore further, control and Cdon‐depleted C2C12 cells were serum starved for 16 h and then treated with bFGF (5 ng/mL) for 5, 10, or 30 min and analyzed for expression of *Fos* and *Egr1* by qRT‐PCR (*Figure* 5D). In line with the results shown in *Figure* 5B, Cdon‐depleted C2C12 cells showed significantly delayed and reduced *Fos* and *Egr1* expression, compared with the control cells. Treatment with bFGF for 2 min greatly elevated active, phosphorylated ERK1/2 (p‐ERK1/2) levels in control cells, and this was further increased at 5 min of treatment (*Figure* 5E). In contrast, Cdon‐depleted cells exhibited starkly attenuated ERK activation in response to bFGF. To assess whether this decrease in ERK activation also occurred in regenerating Cdon‐depleted muscles, mock and tmx‐treated muscles at PID4 were subjected to immunoblotting. The levels of p‐ERK1/2 were substantially reduced in Cdon‐depleted muscles, compared with control muscles (*Figure* 5F and 5G). Collectively, these data demonstrate that the ablation of Cdon in satellite cells impairs ERK activation in response to FGF signalling, likely contributing to defective proliferation and regeneration.

To investigate the potential mechanism leading to the impaired FGF responsiveness in Cdon‐deficient satellite cells, we analyzed a potential interaction between FGFR and Cdon in C2C12 cells by coimmunoprecipitation with control IgG or Cdon antibodies (*Figure* 5H). FGFR1 and FGFR4 proteins were coprecipitated with Cdon in C2C12 cells. Next, we assessed whether Cdon regulates the surface localization of FGFRs by surface biotinylation (*Figure* 5I). Interestingly, the surface levels of FGFR1 and FGFR4 decreased in Cdon‐depleted cells while the surface cadherin level was largely unchanged, relative to the control cells. In a previous study, we demonstrated that Cdon levels were decreased in C2C12 myoblasts cultured on a non‐specific cell adhesion promoting substratum, PLL. Cdon depletion by siRNA or by culture on PLL decreased integrin/FAK/ERK signalling.^26^ To further explore this observation, C2C12 cells were cultured on PLL‐coated or Matrigel‐coated petri dishes to promote integrin signalling in presence of serum. The levels of Cdon, p‐ERK, and pFAK were greatly decreased in cells cultured on PLL while p21 levels were strongly enhanced (*Figure* S12A and S12B). Cells cultured on PLL exhibited starkly blunted proliferation, evident by significantly decreased BrdU incorporation and a concurrent increase in SA‐β‐gal expression, compared with cells cultured on Matrigel (*Figure* S12C and S12D). Similar to Cdon‐depleted cells, C2C12 cells cultured on PLL had diminished surface levels of FGFR1, FGFR4, and Cdon while cadherin levels were largely unchanged (*Figure* 5J). Collectively, these data suggest that Cdon regulates surface localization of FGFR1 and FGFR4, thereby regulating FGF responsiveness of myoblasts.

### Cdon ablation in satellite cells perturbs integrin1 activation

**Figure 6:**
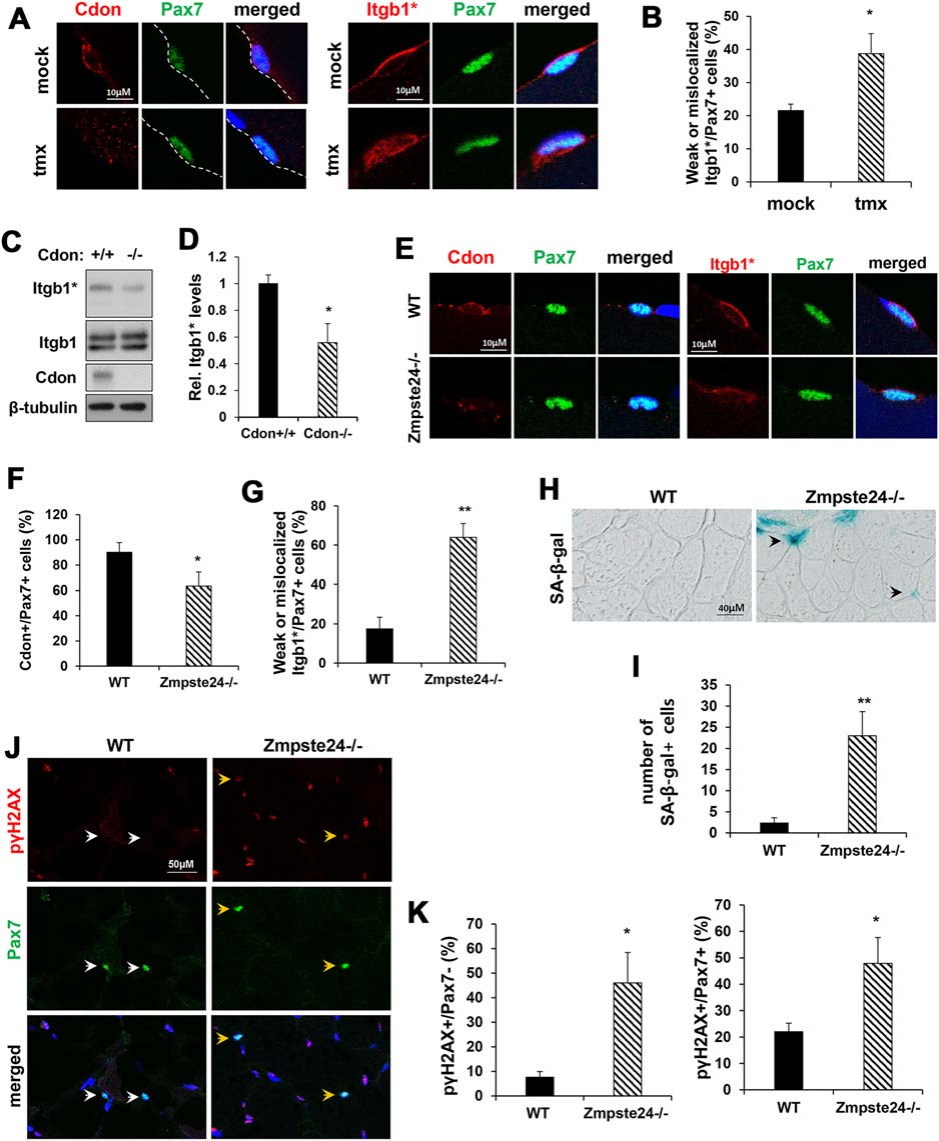
Loss of Cdon results in impaired integrin β1 signalling. (A) Immunostaining for Cdon, activated Itgb1 (Itgb1*) and Pax7 in freshly isolated EDL myofiber. (B) Quantification of Pax7‐positive cells, which have weak or mislocalized Itgb1* (*n* = 3, each group; 8–12 myofibers per animal, *P < 0.05) (C, D) Immunoblot analysis for total Itgb1 and Itgb1* in *Cdon* ^*−/−*^ or *Cdon* ^*+/+*^ primary myoblasts. (*n* = 3, *P < 0.05). (E) Immunostaining for Cdon, activated Itgb1 (Itgb1*) and Pax7 in freshly isolated EDL myofiber of wild type (WT) or *Zmpste24* ^*−/−*^ mice. (F) Quantification of double positive cells for Cdon and Pax7. (*n* = 3, each group; 8–12 myofibers per animal, *P < 0.05) (G) Quantification of Pax7+ cells with decreased or mislocalized Itgb1* (*n* = 3, each group; 8–12 myofibers per animal, **P < 0.01). (H) SA‐β‐gal staining of TA muscles from *Zmpste24* ^*−/−*^ mice. (I) Quantification of SA‐β‐gal‐positive cells in TA muscles from *Zmpste24* ^*−/−*^ and wild type mice. (*n* = 3, SA‐β‐gal‐positive cells in four field per each mice, **P < 0.01) (J) Immunostaining for pγH2AX and Pax7 in TA muscles from wild type and *Zmpste24* ^*−/−*^ mice. (K) Quantification of pγH2AX‐positive cells in the Pax7‐negative or Pax7‐positive populations. (*n* = 3; WT, 214–231 nuclei per sample; *Zmpste24* ^*−/−*^, 192–240 nuclei per sample, *P < 0.05).

A previous study suggested that defective integrin signalling is linked with impaired FGF responsiveness, thereby contributing to reduced proliferation of aged muscle stem cells.^20^ We therefore examined integrin activation in satellite cells on freshly isolated myofibers from vehicle and tmx‐treated mice by immunostaining with antibodies to active integrin β1 (Itgb1*) and Cdon (*Figure* 6A and 6B). About 76% of control Pax7‐positive satellite cells had polarized localization of Itgb1* at the basal lamina contact site of satellite cells. In contrast, only ~40% of Cdon‐depleted satellite cells had nonpolarized Itgb1* proteins, and they frequently had weaker overall Itgb1* signals. This result resembled the phenotype observed in aged satellite cells.^20^ The level of Itgb1* protein was assessed in control and Cdon‐deficient primary myoblasts (*Figure* 6C and 6D). Itgb1* levels decreased in Cdon‐deficient myoblasts, relative to the control myoblasts while total Itgb1 proteins were unchanged. Additionally, we did not observe any coimmunoprecipitation between Cdon and Itgb1 when overexpressed in 293T cells nor did Cdon depletion reduce cell surface levels of Itgb1 in C2C12 cells (*Figure* S13). A reduction in expression of the integrin ligand fibronectin has been implicated in aged satellite cell function.^22^ However, the RNA sequencing data revealed that Fibronectin expression was not altered in Cdon‐depleted muscles whereas distinct collagen types (Col1a1, Col5a1, Col3a1, and Col14a1) were significantly altered, likely reflecting the enhanced fibrosis in Cdon‐depleted muscles (*Figure* S10).

To further understand the role of Cdon in muscle aging, we examined expression of Cdon and Itgb1* in satellite cells from mice with the Zmpste24 protease deficiency, of a model for progeria.^39^ These mice exhibit, among other aging‐related phenotypes, premature muscle loss associated with elevated cellular senescence and reduced proliferation of muscle stem cells.^40^, ^41^, ^42^ Myofibers isolated from EDL muscles of 4‐month‐old wild type or *Zmpste24* ^*−/−*^ mice were immunostained for Cdon, Itgb1*, and Pax7 (*Figure* 6E–6G). About 90% of Pax7‐positive satellite cells from control myofibers were positive for Cdon expression, while about 63% of Pax7‐positive cells from Zmpste24‐deficient myofibers were positive for Cdon. Similarly to Cdon‐deficient satellite cells, more Zmpste24‐deficient satellite cells exhibited either weaker or nonpolarized Itgb1* signal, compared with the wild type cells. These data collectively suggest that Cdon and integrin signalling are perturbed in aging satellite cells. Furthermore, SA‐β‐gal‐positive cells were easily observed in Zmpste24‐deficient muscles while wild type muscles had only very few cells positive for SA‐β‐gal‐positive cells (*Figure* 6H and 6I). Additionally, Zmpste24‐deficient muscles also exhibited greatly elevated immunostaining for pγH2AX in both Pax7‐positive and negative cells compared with wild type muscles (*Figure* 6J and 6K). These results suggested that Cdon depletion is correlated with satellite cell aging. In summary, our study demonstrates a key role of Cdon in the maintenance of satellite cell self‐renewal and proliferation through FGFR trafficking and signalling.

## Discussion

In the current study, we determined the role of Cdon in satellite cells by using inducible Pax7‐CreERT2‐mediated ablation in mice. Cdon is expressed in Pax7‐positive satellite cells and more frequently localized basally, similar to Itgb1* proteins. Satellite cell‐specific deletion of Cdon led to impaired, fibrotic muscle regeneration, likely attributable to diminished proliferation and cellular senescence of satellite cells; 4 days after CTX injury, we did not observe differences in expression of myogenic regulators, including Myf5, MyoD, and Mef2c, between control and Cdon‐deficient satellite cells, suggesting no significant defects in satellite cell activation. In contrast, we detected major alterations in genes related to proliferation, inflammation, and extracellular matrices in Cdon‐deficient muscles. This alteration in the transcriptome is well aligned with the decreased proliferation of muscle progenitor cells, impaired muscle regeneration, and increased fibrosis observed in Cdon‐depleted muscles.

Regulation and strength of cell adhesion signalling, including integrin and cadherin signalling, is essential to control of quiescence and proliferation of satellite cells.^20^, ^21^ Loss of N‐cadherin and M‐cadherin in satellite cells leads to a state of partial activation and long‐term expansion of a regeneration‐proficient satellite cell pool.^21^ Alteration in integrin signalling causes reduced responsiveness to FGF, thereby reducing the proliferative capacity of satellite cells.^20^ Considering the relationship of Cdon with cell adhesion signalling, especially the physical interaction of Cdon with N‐cadherin and M‐cadherin,^23^ we predicted a function for Cdon similar to cadherins in the control of satellite cell quiescence and activation. Therefore, the difference in their phenotypes was surprising. One possible mechanism might be distinct cellular localizations in satellite cells. Cadherins are localized at the myofiber contact side of satellite cells, while Cdon is found more often at the basal lamina contact side, similarly to Itgb1*. This localization appears to be important because Cdon‐depleted satellite cells showed aberrant regulation of Itgb1*. Consistent with this, satellite cells from Zmpste24‐deficient progeric mice also exhibited aberrant Itgb1* localization and Cdon expression. It was shown previously that Zmpste24‐deficiency causes cellular senescence and decreased proliferation of muscle progenitor cells,^39^, ^41^, ^42^ resembling the phenotype of Cdon deficiency. Therefore, aberrant integrin β1 signalling may contribute to the impaired FGF responsiveness of Cdon‐depleted satellite cells. Another mechanism for impaired responsiveness to bFGF is likely to be reduction in cell surface FGFR1 and four levels in Cdon‐depleted myoblasts. The physical interaction of FGFR1/4 and Cdon resembles the interaction of FGFR with other immunoglobulin superfamily members like N‐CAM and L1 in the control of neurite outgrowth.^43^ This interaction is mediated through a cell adhesion molecule‐homology domain of these molecules.^43^ Although Cdon shares ectodomain structural homology with N‐CAM and L1, their sequences are not well‐conserved at the amino acid level.^44^, ^45^

The deletion of Itgb1 in satellite cells caused impaired muscle regeneration via defects in self‐renewal and proliferation of satellite cells.^20^ Furthermore, aged satellite cells exhibited aberrant Itgb1* localization with abrogated FGF responsiveness.^20^ Cdon ablation caused defects similar to those seen in Itgb1‐ablated satellite cells, and aberrant Itgb1 activation was observed in Cdon‐ablated cells without affecting Itgb1 cell surface levels. Although Cdon and Itgb1 colocalized at the ECM contact site, we failed to detect a physical interaction between Cdon and Itgb1. It is conceivable that Cdon regulates the surface localization of FGFR in the vicinity of Itgb1 at the ECM contact site, leading to efficient FGFR signalling. Similar to Cdon‐depleted myoblasts, C2C12 cells cultured on non‐integrin‐binding PLL matrix had impaired proliferation and senescence. Thus, it is likely that crosstalk between Cdon and integrin signalling is important for the proliferation of myoblasts, and Cdon deficiency likely causes impaired proliferation of satellite cells via aberrant integrin and FGFR signalling.

Interestingly, Cdon depletion in satellite cells did not cause overt cell death while it elevated cellular stress and senescence, as seen by the expression of p21, SASPs, and pγH2AX. Because the above mentioned pathways are not directly implicated in cellular senescence, Cdon likely regulates other pathways also, and further study will be required to identify the direct trigger for cellular senescence in Cdon‐depleted cells. In summary, we have demonstrated that Cdon is a critical factor in regulation of integrin and FGFR signalling and thereby the proliferative capacity of satellite cells.

## Author contributions

J. H. B., M. H., H. J. J., H. B. K., R. S. K., and J. S. K. developed the study concept and design. J. H. B. M. H., H. J. J., H. B. K., S. J. L., M. H., Y. S. L., and S. C. J. performed experiments and collected data. J. H. B., H. J. J., D. R. R., Y. S. L., and J. S. K. interpreted data, prepared the figures. J. H. B., H. J. J., G. U. B., R. S. K., and J. S. K. wrote the manuscript.

## Conflict of interest

Ju‐Hyeon Bae, Mingi Hong, Hyeon‐Ju Jeong, Hye‐Been Kim, Sang‐Jin Lee, Dongryeol Ryu, Gyu‐Un Bae, Sung Chun Cho, Young‐Sam Lee, Robert S. Krauss, and Jong‐Sun Kang declare that they have no conflict of interest.

## Supporting information


**Figure S1.** (A) Scheme for derivation of a conditional *Cdon* knockout allele. *Cdon* ^*tm3a*^ is a targeted trap allele with a *lacZ* reporter‐tagged insertion. *Cdon* ^*tm3b*^ is a *lacZ*‐tagged knockout allele obtained after crossing *Cdon* ^*tm3a*^ mice with mice expressing Cre recombinase. Exon 9 is deleted leading to a truncated Cdon‐lacZ fusion protein. *Cdon* ^*tm3c*^ is a conditional allele with loxP sites flanking Exon 9, obtained by crossing *Cdon* ^*tm3a*^ mice with mice expressing Flp recombinase. For this study, *Cdon* ^*tm3c*^ mice were generated by crossing *Cdon* ^*tm3a*^ mice with ROSA‐Flpo mice (kindly provided by Phil Soriano). *Cdon* ^*tm3d*^ is a knockout allele obtained by crossing *Cdon* ^*tm3c*^ mice with mice expressing Cre recombinase, and is referred to in this manuscript as *Cdo* ^*f*^. For this study, *Cdon* ^*tm3c*^ mice were crossed with *Pax7* ^*CreERT2*^ mice, to generate the *Cdon* ^*tm3d*^ genotype specifically in satellite cells. Bold black lines indicate probes used on Southern blots. The predicted Cdon protein from each allele is in green, with or without lacZ (in blue). Yellow boxes represent exons, except the floxed exon 9, which is in orange. Arrows represent primers for screening and genotyping. Primers are: Cdon5: TAGCTTCCCAGAGGGTGTGAGAGC; Cdon3: ATGCTGACATTAGGAGCAAATGCG; LAR3: CAACGGGTTCTTCTGTTAGTCC; CdonF: CCTGGGTATGTGTGAGACATTTGC; loxR: TGAACTGATGGCGAGCTCAGACC. (B) Southern blot analysis. Genomic DNA from the ES cells was digested with NsiI for detection of the recombined 5’ arm and NheI for detection of the recombined 3’ arm. Wt and mutant bands are indicated by arrows. (C) PCR genotyping of each allele. Primers used for each PCR and product size: *Cdon* ^*tm3a*^: Cdon5, Cdon3, and LAR3 (wt band is 517 bp, *Cdon* ^*tm3a*^ band is 330 bp); *Cdon* ^*tm3c*^: Cdon5 and Cdon3 (wt band is 494 bp, *Cdon* ^*tm3*^ band is 603 bp); *Cdon* ^*tm3d*^: Cdon5, Cdon3, and loxR (wt band is 517 bp, *Cdon* ^*tm3d*^ band is 266 bp). (D) Western blot analysis of the conditional knockout allele, *Cdon* ^*tm3d*^. *Cdon* ^*tm3c*^ was crossed to *Meox2*‐cre mice (which express Cre in the epiblast; kindly provided by Phil Soriano) to generate *Cdon* ^*tm3d*^ mice. Western blot of two wt and ten *Cdon* ^*tm3d*^ embryos. Whole embryo lysates were prepared from four different litters harvested at E10.5 were probed with antibodies to Cdon (R&D Systems) and as a control‐actin (Abcam).

**Figure S2.** Cdon (red) and Pax7 (green) immunostaining of satellite cells on single myofibers isolated from EDL muscles. DAPI labels nuclei (blue). Cdon localization in individual Pax7+ cells was quantified and shown in the pie chart as no signal or the site of predominant localization in the whole membrane, basal membrane, or apical membrane. 8‐12 myofibers per EDL muscle from three 4‐month‐old mice were used for immunostaining and total 66 pax7‐positive cells were quantified.

**Figure S3.** (A) Immunoblot of Cdon depletion (B) Quantification of Cdon protein levels in regenerating muscles after Cdon ablation by tamoxifen treatment. (n = 3, *p < 0.05) (C) Quantitative RT‐PCR for Cdon. Satellite cells and fibroblasts were isolated from hindlimbs of *Cdon* ^*fl/fl*^ *;Pax7* ^*CreERT2*^ mice. (n = 3, ***p < 0.001).

**Figure S4.** (A) Histological analysis (hematoxylin and eosin, H&E) of mock or tmx‐treated TA muscles from 21 days post the first injury. (B) Quantification of myofiber size. (n = 3, **p < 0.01, ***p < 0.001).

**Figure S5.** Weights of TA muscles of mock‐ or tmx‐treated *Cdon* ^*fl/fl*^ *;Pax7* ^*CreERT2*^ mice at PID7 or PID21. (n = 3, *p < 0.05).

**Figure S6.** (A, B) TA muscles at 4 days post the first injury were immunostained for Pax7 (green) and Ki67 (red). Nuclei were visualized by DAPI staining (blue). Quantification of Pax7 and Ki67‐ double positive cells and the values presented as percentile relative to total Pax7‐positive cells. Total Pax7‐positive cells counted were 693 for mock and 579 for tmx muscles. (n = 3, *p < 0.05).

**Figure S7**. Immunostaining for cleaved‐Caspase 3 in *Cdon* ^*+/+*^ and *Cdon* ^*‐/‐*^ myoblasts. As a control, cell death was induced by treatment with 1 M staurosporin for 3 hours, n = 5.

**Figure S8**. Immunostaining for pγH2AX in *Cdon* ^*+/+*^ and *Cdon* ^*‐/‐*^ myoblasts. (n = 5, ***p < 0.001).

**Figure S9**. Top 10 list for enriched GO terms based on biological function (A), KEGG pathway (B) or GO terms on cellular component (C). (*p < 0.05, FDR *q* value <0.05).

**Figure S10**. Heat maps represent statistically significant gene lists involved in inflammatory response, extracellular matrix, and negative regulation of cell population proliferation that are up‐ (red) or down‐regulated (green) in tmx‐treated muscle. (Fold change (FC) ≥ 1.5 or ≤ 0.666, *p < 0.05).

**Figure S11**. (A, B) Volcano plot for representing 877 statistically significant genes. (Fold change (FC) ≥ 1.5 or ≤ 0.666, *p < 0.05). Upregulated genes in tmx‐treated muscles are labelled as red while downregulated genes are labelled as green, grey represents other genes, including Muscle regulatory factors (MRFs), Fibroblast growth factors (Fgfs), Fibroblast growth factor receptors (Fgfrs) and Hepatocyte growth factor (Hgf).

**Figure S12.** (A) Immunoblot analysis for Cdon, ERK2, pERK1/2, pFAK, FAK, and p21 in C2C12 cells which were grown on Matrigel‐ or poly‐L‐lysine‐coated Petri dishes. (B) Fold‐change from panel A. pERK or pFAK were normalized by levels of ERK2 or FAK, respectively. The value of the control muscle was set to 1. (n = 3, **p < 0.01, ***p < 0.001). (C, D) SA‐β‐gal and BrdU staining of C2C12 myoblasts (n = 3, ***p < 0.001).

**Figure S13.** (A) Lysates of 293 T cells transfected with control, Itgb1, and/or Cdon vectors as indicated were subjected to immunoprecipitation with Cdon antibodies and immunoblotting. (B) Control and Cdon‐depleted C2C12 cells were subjected to biotinylation and pulldown with streptavidin bead followed by immunoblotting for Itgb1 and Cadherin.

**Table S1.** List of primer sequences

**Table S2.** List of antibodies used in this body
