# Corrigendum to Bae et al. *J Cachexia Sarcopenia Muscle* 11, 1089–1103, 2020. doi: 10.1002/jcsm.12563

**DOI:** 10.1002/jcsm.12628

**Published:** 2020-10-15

In *Figure* S1A of the original manuscript, it was indicated that exon 9 of the *Cdon* locus was flanked by *loxP* sites. In fact, it is exon 8 that is flanked by *loxP* sites. A revised figure with this correction has been provided.


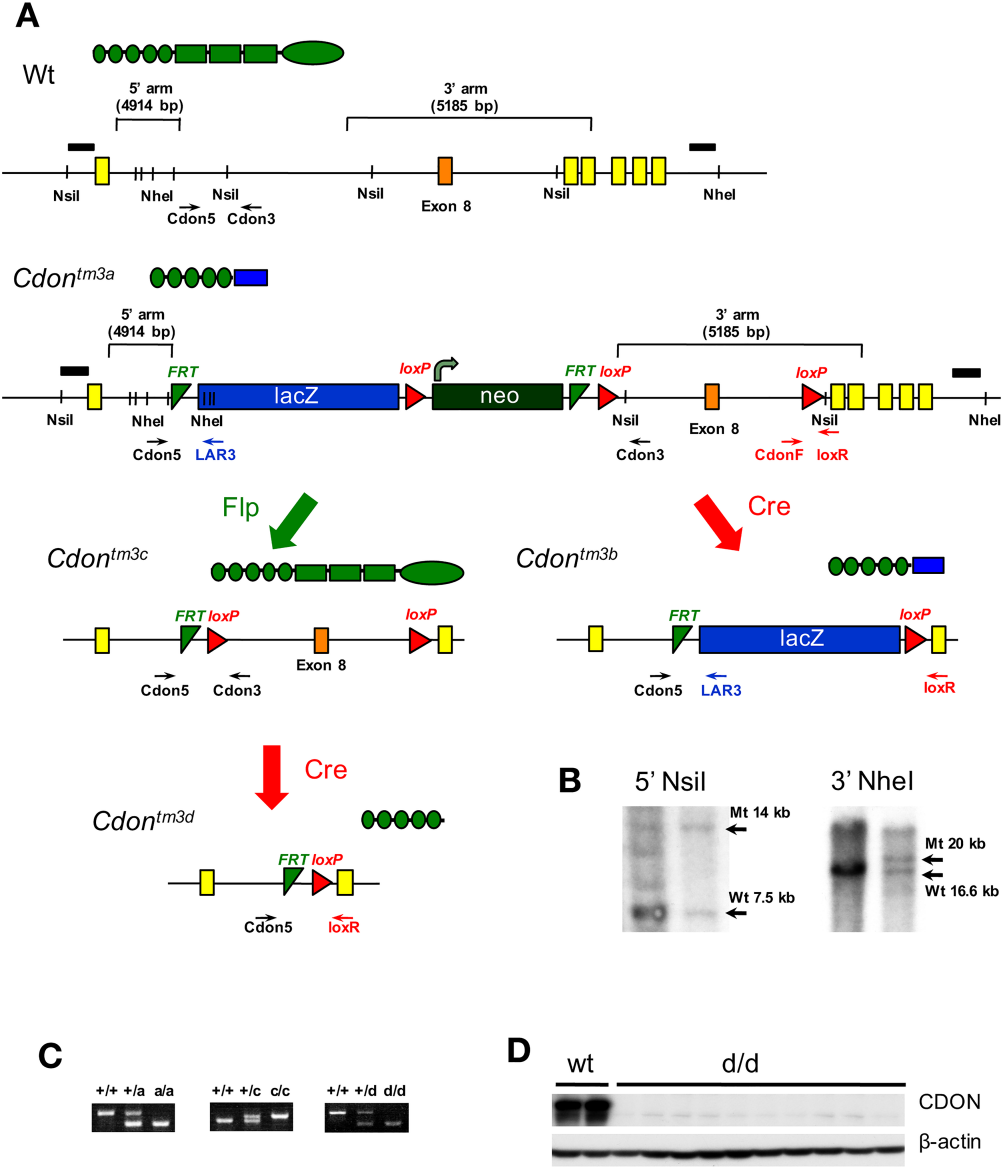


## Supporting information


**Figure S1.** Supporting Information

