# Supplementary figures and images for: Satellite cell‐specific ablation of Cdon impairs integrin activation, FGF signalling, and muscle regeneration
Source: J Cachexia Sarcopenia Muscle. 2020 Feb 27;11(4):1089–103. doi: 10.1002/jcsm.12563 (PMC7432598; doi:10.1002/jcsm.12563)

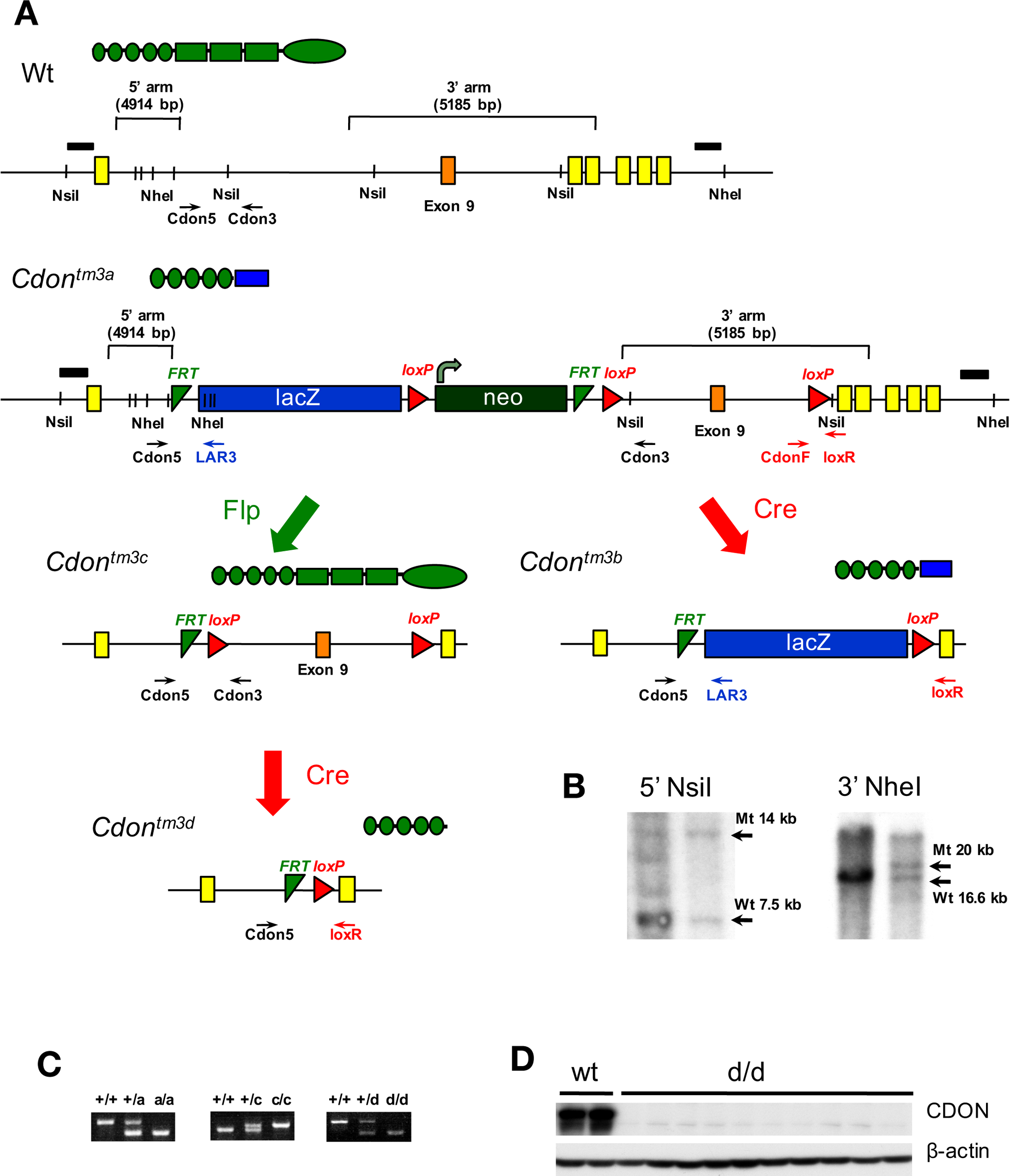

Supplement: Supplementary file 1 — Figure S1. (A) Scheme for derivation of a conditional Cdon knockout allele. Cdon tm3a is a targeted trap allele with a lacZ reporter‐tagged insertion. Cdon tm3b is a lacZ‐tagged knockout allele obtained after crossing Cdon tm3a mice with mice expressing Cre recombinase. Exon 9 is deleted leading to a truncated Cdon‐lacZ fusion protein. Cdon tm3c is a conditional allele with loxP sites flanking Exon 9, obtained by crossing Cdon tm3a mice with mice expressing Flp recombinase. For this study, Cdon tm3c mice were generated by crossing Cdon tm3a mice with ROSA‐Flpo mice (kindly provided by Phil Soriano). Cdon tm3d is a knockout allele obtained by crossing Cdon tm3c mice with mice expressing Cre recombinase, and is referred to in this manuscript as Cdo f. For this study, Cdon tm3c mice were crossed with Pax7 CreERT2 mice, to generate the Cdon tm3d genotype specifically in satellite cells. Bold black lines indicate probes used on Southern blots. The predicted Cdon protein from each allele is in green, with or without lacZ (in blue). Yellow boxes represent exons, except the floxed exon 9, which is in orange. Arrows represent primers for screening and genotyping. Primers are: Cdon5: TAGCTTCCCAGAGGGTGTGAGAGC; Cdon3: ATGCTGACATTAGGAGCAAATGCG; LAR3: CAACGGGTTCTTCTGTTAGTCC; CdonF: CCTGGGTATGTGTGAGACATTTGC; loxR: TGAACTGATGGCGAGCTCAGACC. (B) Southern blot analysis. Genomic DNA from the ES cells was digested with NsiI for detection of the recombined 5’ arm and NheI for detection of the recombined 3’ arm. Wt and mutant bands are indicated by arrows. (C) PCR genotyping of each allele. Primers used for each PCR and product size: Cdon tm3a: Cdon5, Cdon3, and LAR3 (wt band is 517 bp, Cdon tm3a band is 330 bp); Cdon tm3c: Cdon5 and Cdon3 (wt band is 494 bp, Cdon tm3 band is 603 bp); Cdon tm3d: Cdon5, Cdon3, and loxR (wt band is 517 bp, Cdon tm3d band is 266 bp). (D) Western blot analysis of the conditional knockout allele, Cdon tm3d. Cdon tm3c was crossed to Meox2‐cre mice (which expres [file JCSM-11-1089-s001.tif]

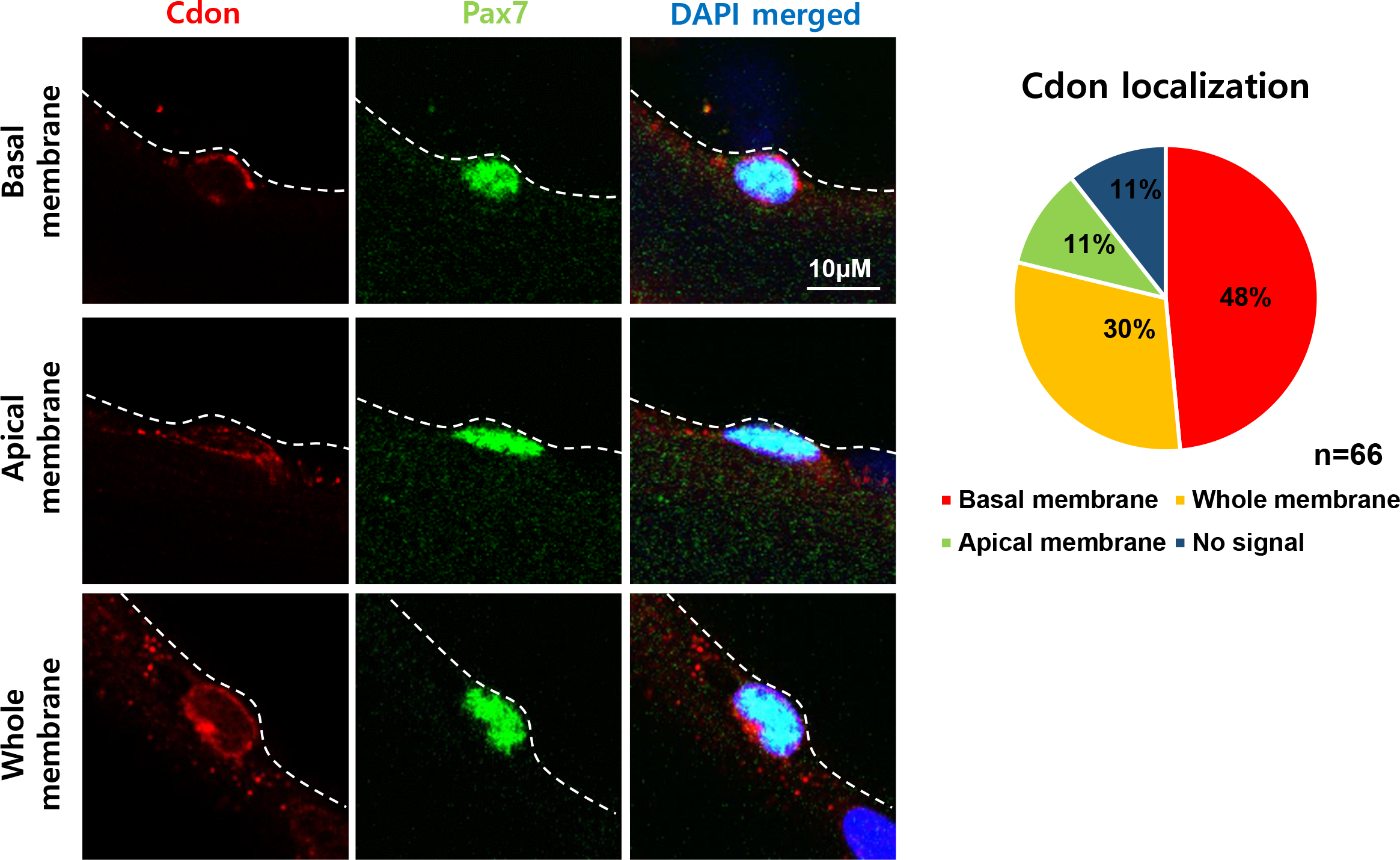

Supplement: Supplementary file 2 — Figure S2. Cdon (red) and Pax7 (green) immunostaining of satellite cells on single myofibers isolated from EDL muscles. DAPI labels nuclei (blue). Cdon localization in individual Pax7+ cells was quantified and shown in the pie chart as no signal or the site of predominant localization in the whole membrane, basal membrane, or apical membrane. 8‐12 myofibers per EDL muscle from three 4‐month‐old mice were used for immunostaining and total 66 pax7‐positive cells were quantified. [file JCSM-11-1089-s008.tif]

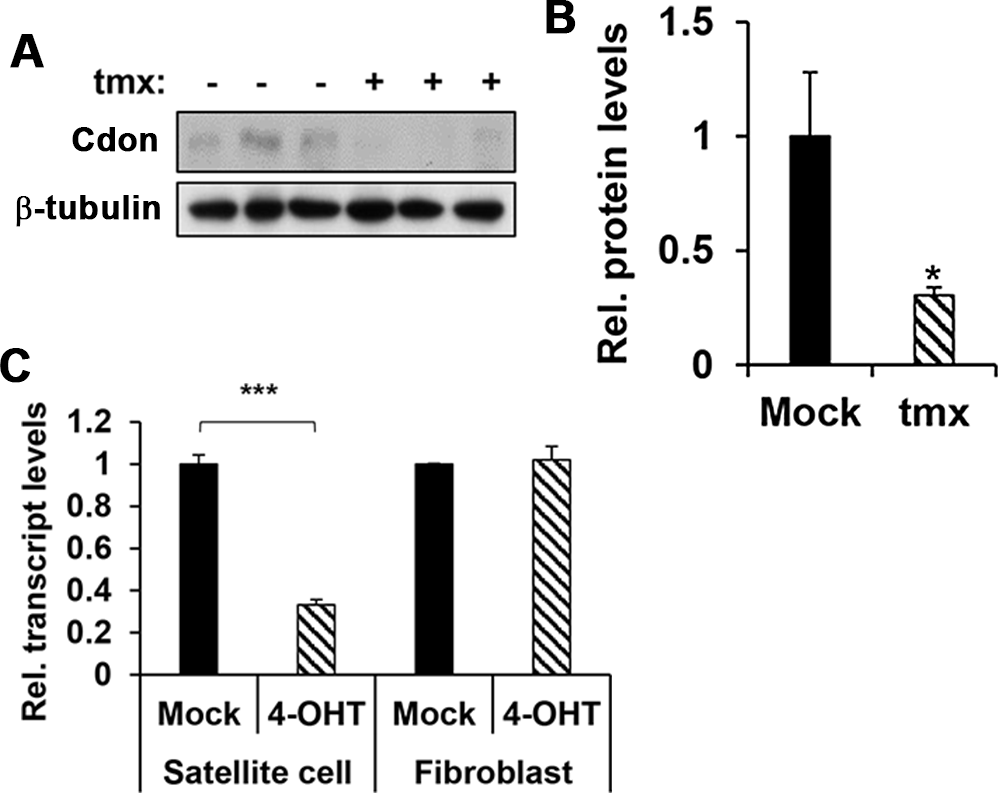

Supplement: Supplementary file 3 — Figure S3. (A) Immunoblot of Cdon depletion (B) Quantification of Cdon protein levels in regenerating muscles after Cdon ablation by tamoxifen treatment. (n = 3, *p < 0.05) (C) Quantitative RT‐PCR for Cdon. Satellite cells and fibroblasts were isolated from hindlimbs of Cdon fl/fl ;Pax7 CreERT2 mice. (n = 3, ***p < 0.001). [file JCSM-11-1089-s009.tif]

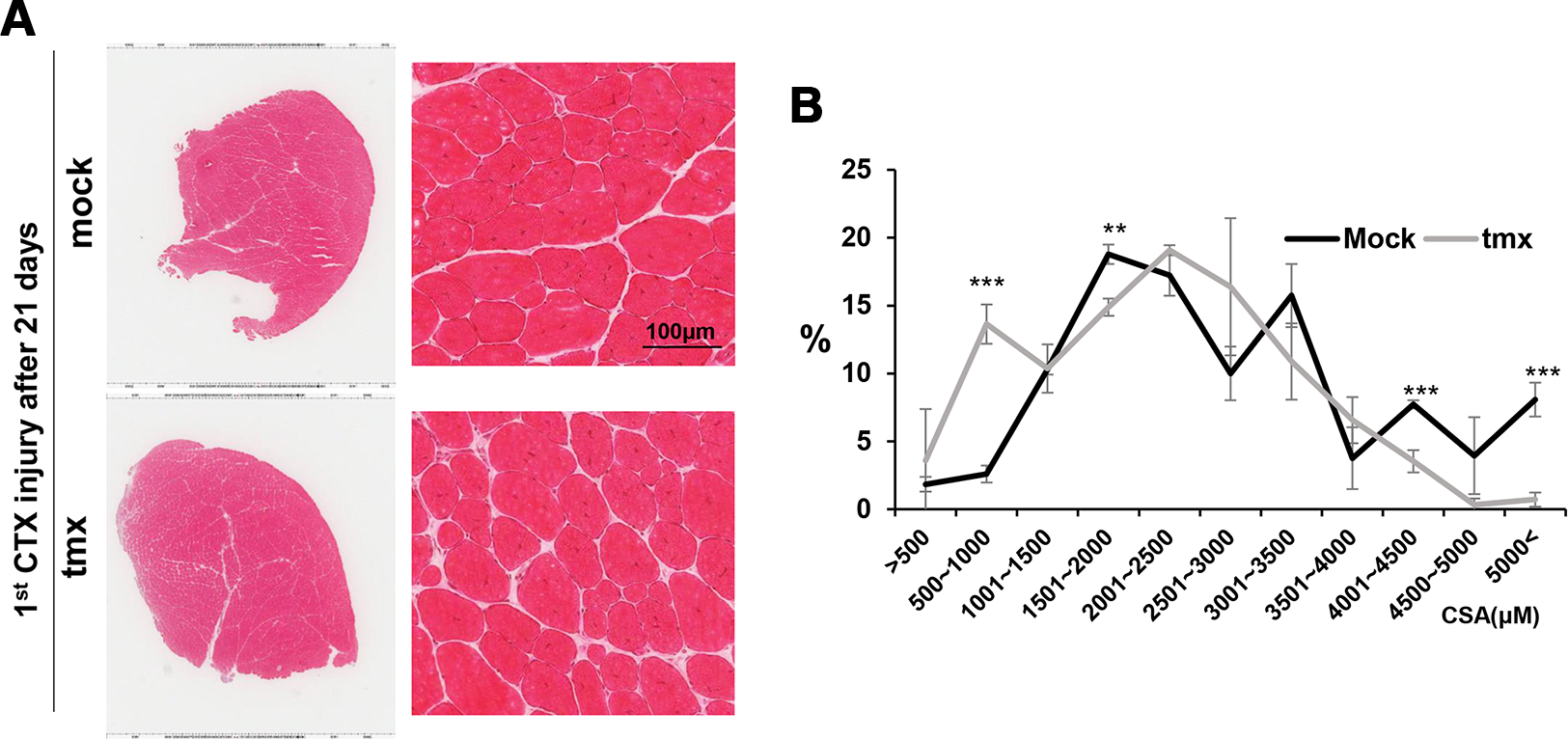

Supplement: Supplementary file 4 — Figure S4. (A) Histological analysis (hematoxylin and eosin, H&E) of mock or tmx‐treated TA muscles from 21 days post the first injury. (B) Quantification of myofiber size. (n = 3, **p < 0.01, ***p < 0.001). [file JCSM-11-1089-s010.tif]

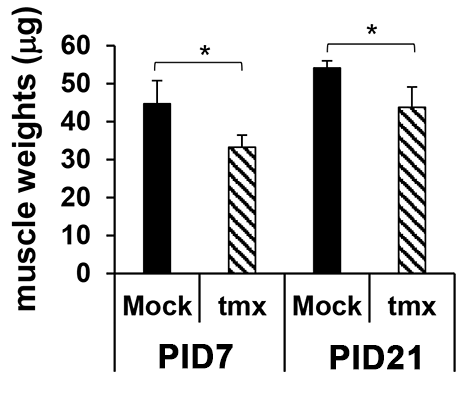

Supplement: Supplementary file 5 — Figure S5. Weights of TA muscles of mock‐ or tmx‐treated Cdon fl/fl ;Pax7 CreERT2 mice at PID7 or PID21. (n = 3, *p < 0.05). [file JCSM-11-1089-s011.tif]

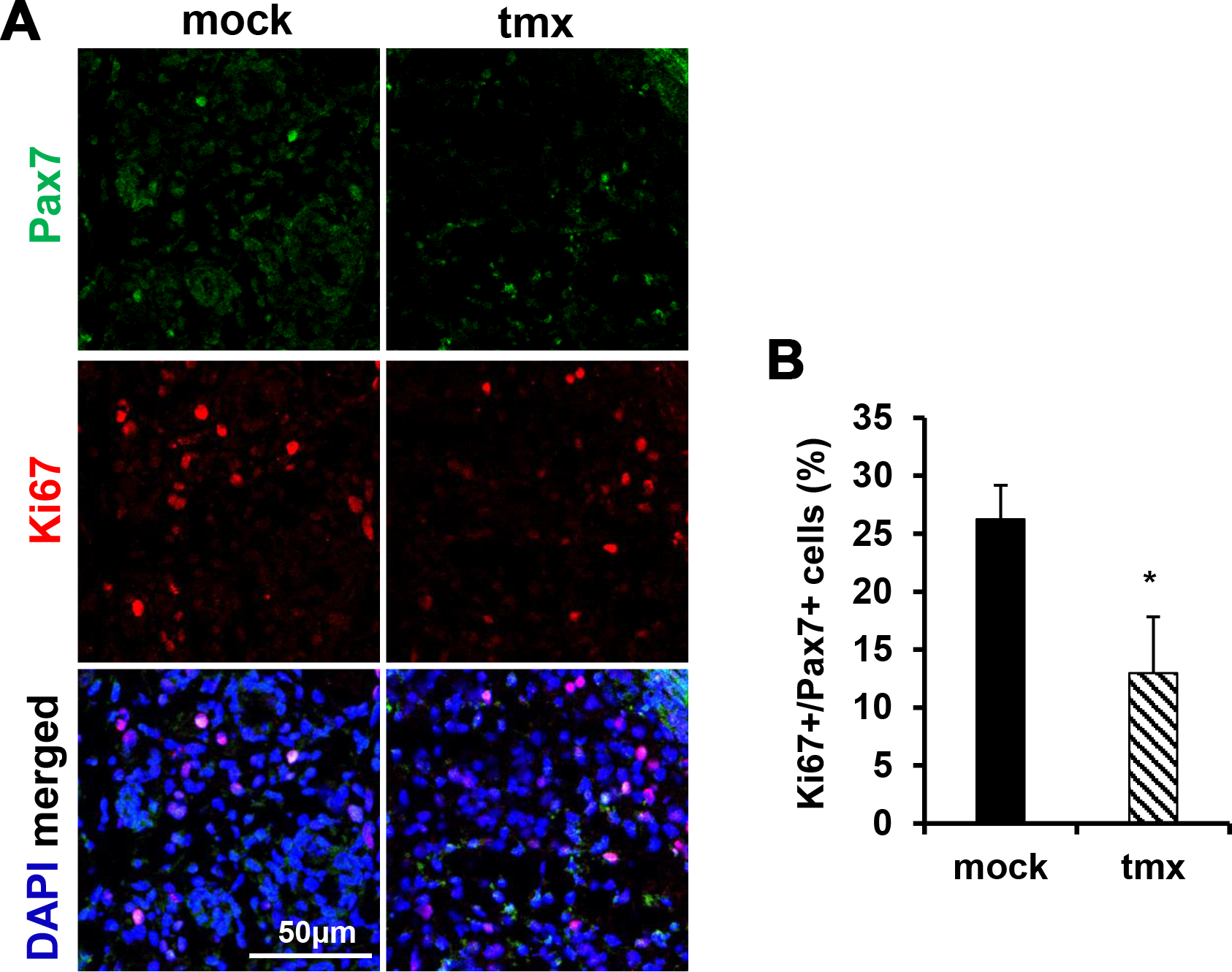

Supplement: Supplementary file 6 — Figure S6. (A, B) TA muscles at 4 days post the first injury were immunostained for Pax7 (green) and Ki67 (red). Nuclei were visualized by DAPI staining (blue). Quantification of Pax7 and Ki67‐ double positive cells and the values presented as percentile relative to total Pax7‐positive cells. Total Pax7‐positive cells counted were 693 for mock and 579 for tmx muscles. (n = 3, *p < 0.05). [file JCSM-11-1089-s012.tif]

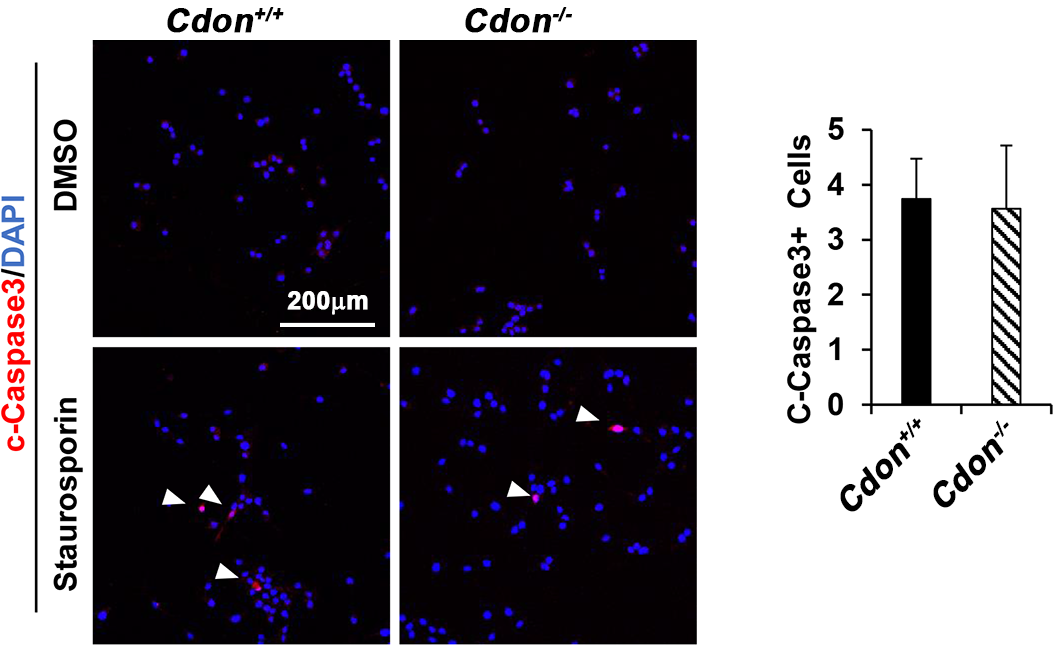

Supplement: Supplementary file 7 — Figure S7. Immunostaining for cleaved‐Caspase 3 in Cdon +/+ and Cdon ‐/‐ myoblasts. As a control, cell death was induced by treatment with 1 M staurosporin for 3 hours, n = 5. [file JCSM-11-1089-s013.tif]

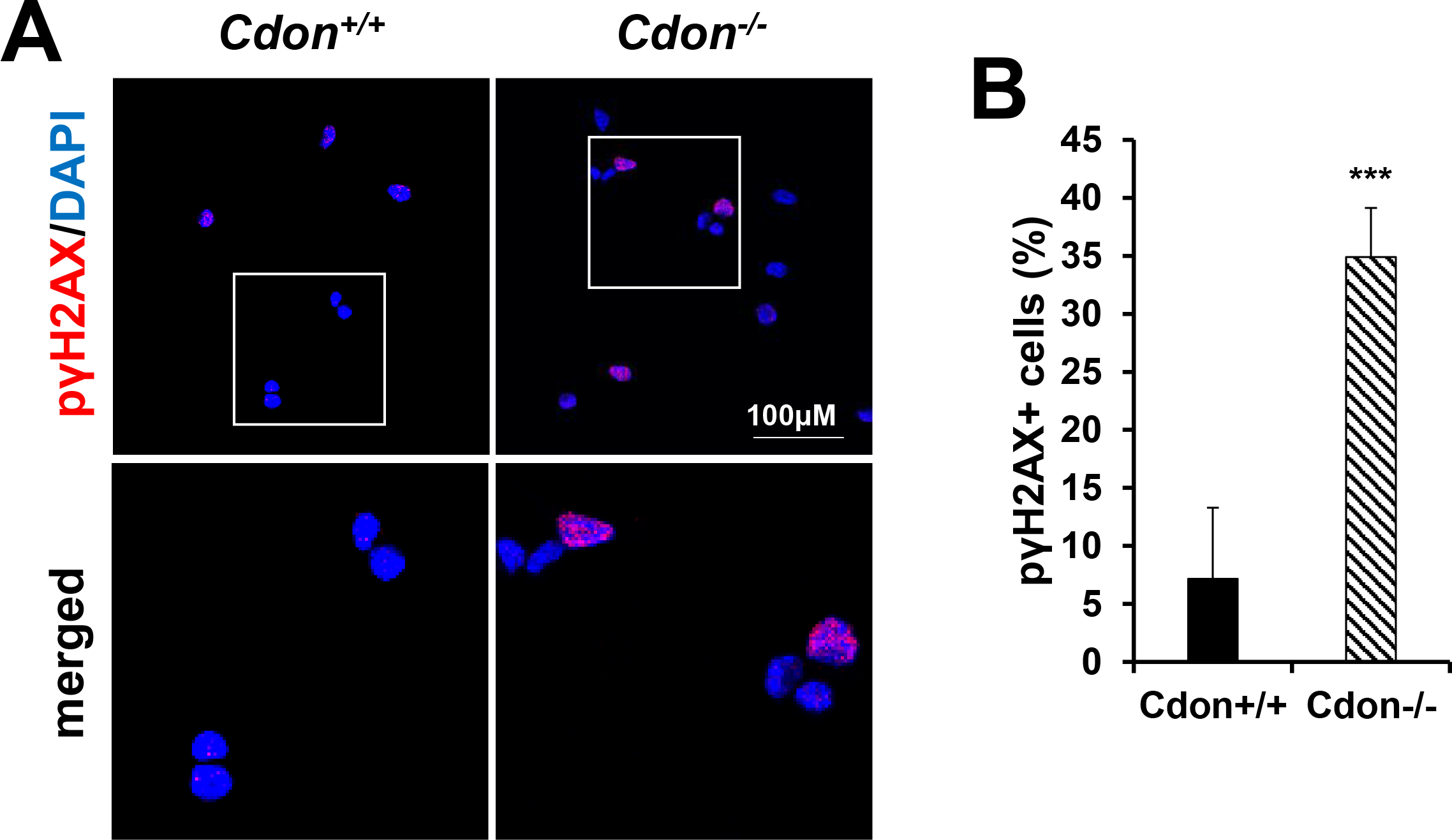

Supplement: Supplementary file 8 — Figure S8. Immunostaining for pγH2AX in Cdon +/+ and Cdon ‐/‐ myoblasts. (n = 5, ***p < 0.001). [file JCSM-11-1089-s014.tif]

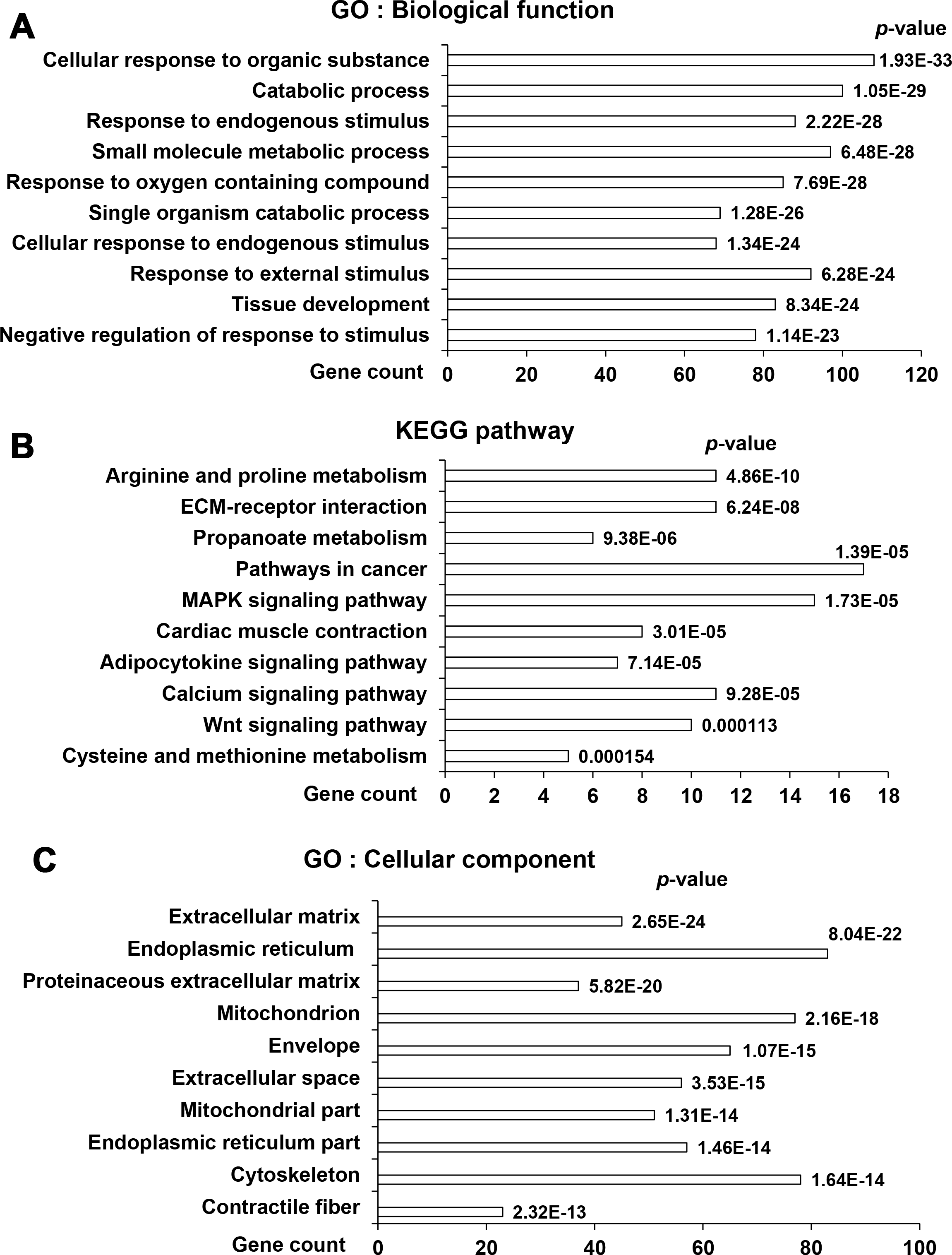

Supplement: Supplementary file 9 — Figure S9. Top 10 list for enriched GO terms based on biological function (A), KEGG pathway (B) or GO terms on cellular component (C). (*p < 0.05, FDR q value <0.05). [file JCSM-11-1089-s015.tif]

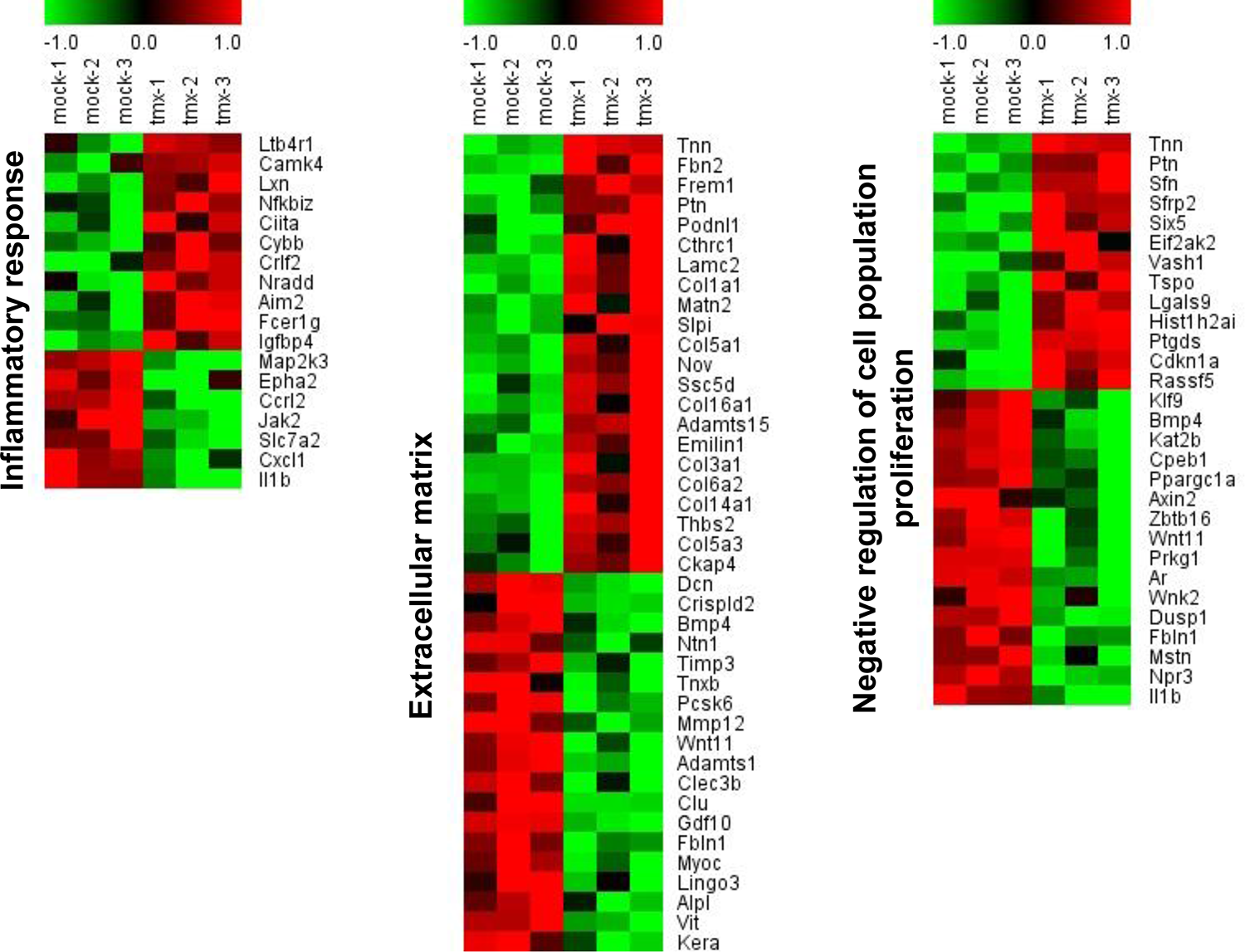

Supplement: Supplementary file 10 — Figure S10. Heat maps represent statistically significant gene lists involved in inflammatory response, extracellular matrix, and negative regulation of cell population proliferation that are up‐ (red) or down‐regulated (green) in tmx‐treated muscle. (Fold change (FC) ≥ 1.5 or ≤ 0.666, *p < 0.05). [file JCSM-11-1089-s002.tif]

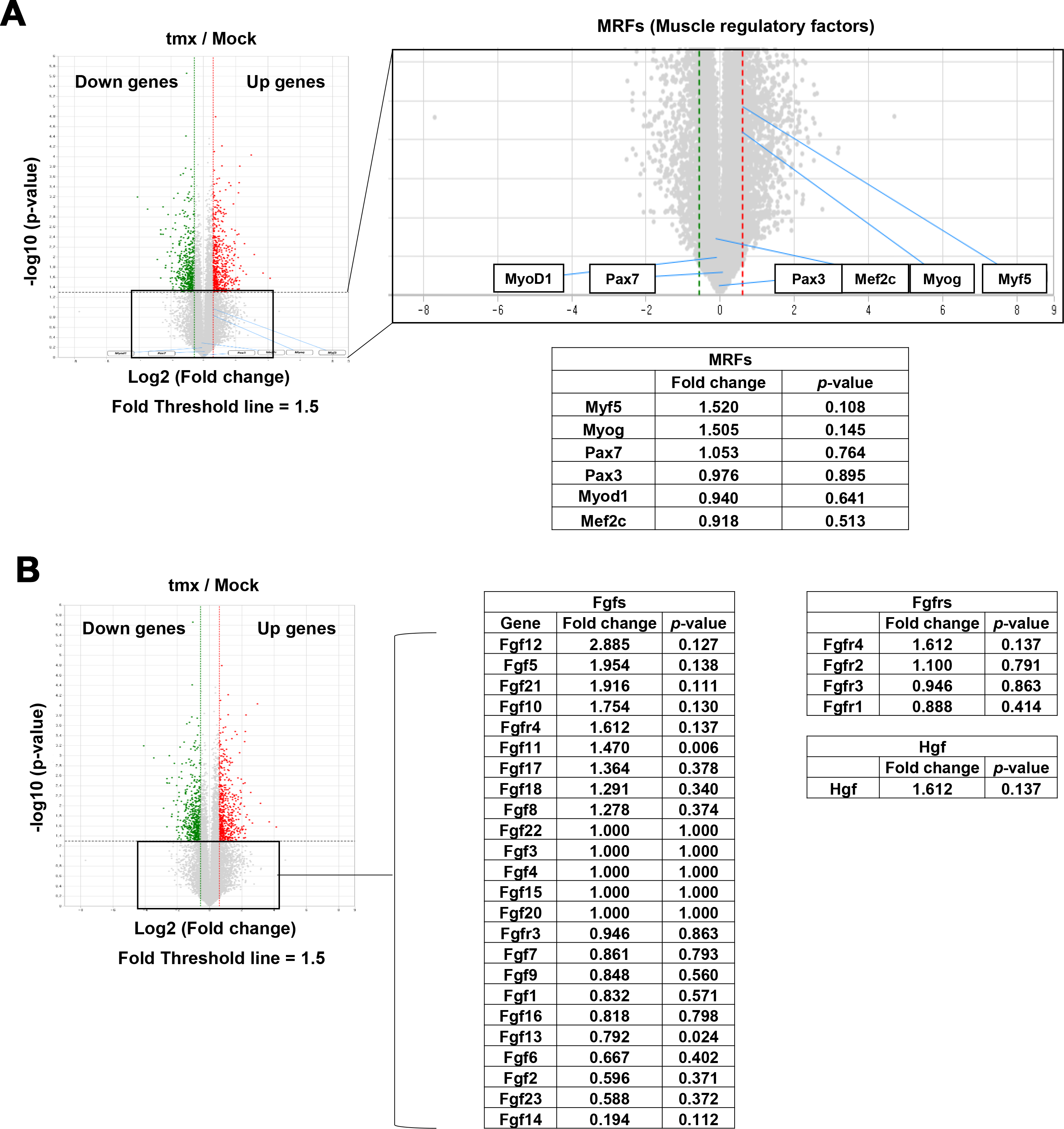

Supplement: Supplementary file 11 — Figure S11. (A, B) Volcano plot for representing 877 statistically significant genes. (Fold change (FC) ≥ 1.5 or ≤ 0.666, *p < 0.05). Upregulated genes in tmx‐treated muscles are labelled as red while downregulated genes are labelled as green, grey represents other genes, including Muscle regulatory factors (MRFs), Fibroblast growth factors (Fgfs), Fibroblast growth factor receptors (Fgfrs) and Hepatocyte growth factor (Hgf). [file JCSM-11-1089-s003.tif]

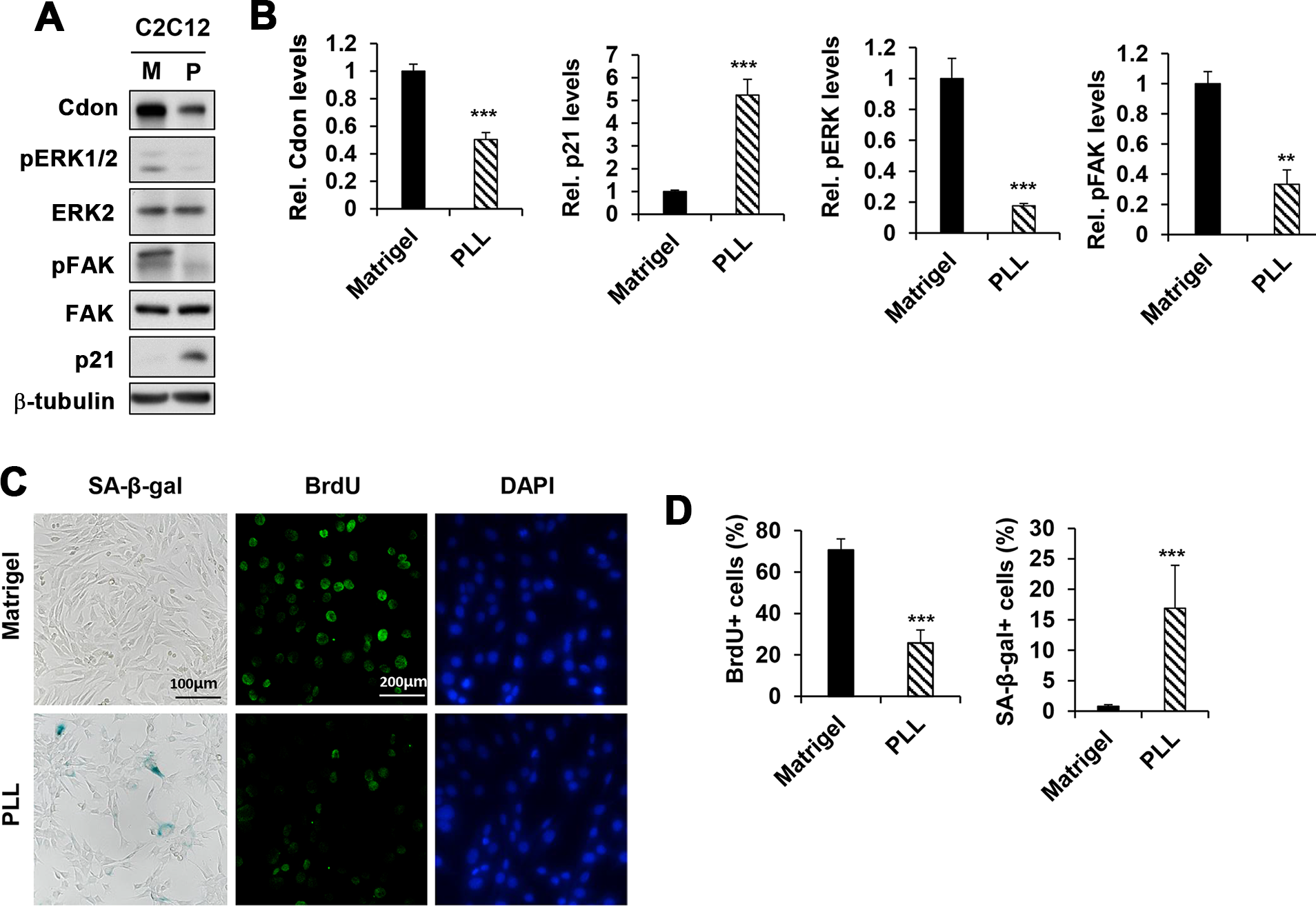

Supplement: Supplementary file 12 — Figure S12. (A) Immunoblot analysis for Cdon, ERK2, pERK1/2, pFAK, FAK, and p21 in C2C12 cells which were grown on Matrigel‐ or poly‐L‐lysine‐coated Petri dishes. (B) Fold‐change from panel A. pERK or pFAK were normalized by levels of ERK2 or FAK, respectively. The value of the control muscle was set to 1. (n = 3, **p < 0.01, ***p < 0.001). (C, D) SA‐β‐gal and BrdU staining of C2C12 myoblasts (n = 3, ***p < 0.001). [file JCSM-11-1089-s004.tif]

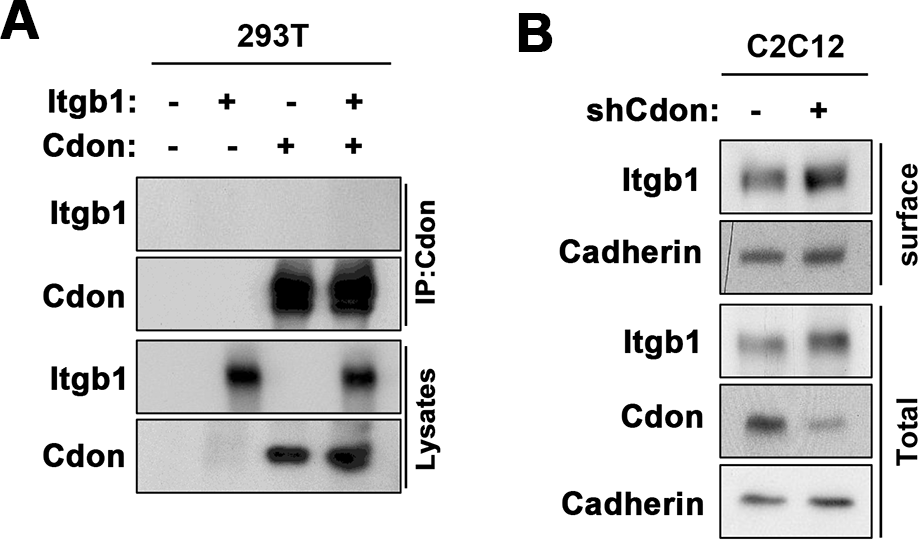

Supplement: Supplementary file 13 — Figure S13. (A) Lysates of 293 T cells transfected with control, Itgb1, and/or Cdon vectors as indicated were subjected to immunoprecipitation with Cdon antibodies and immunoblotting. (B) Control and Cdon‐depleted C2C12 cells were subjected to biotinylation and pulldown with streptavidin bead followed by immunoblotting for Itgb1 and Cadherin. [file JCSM-11-1089-s005.tif]

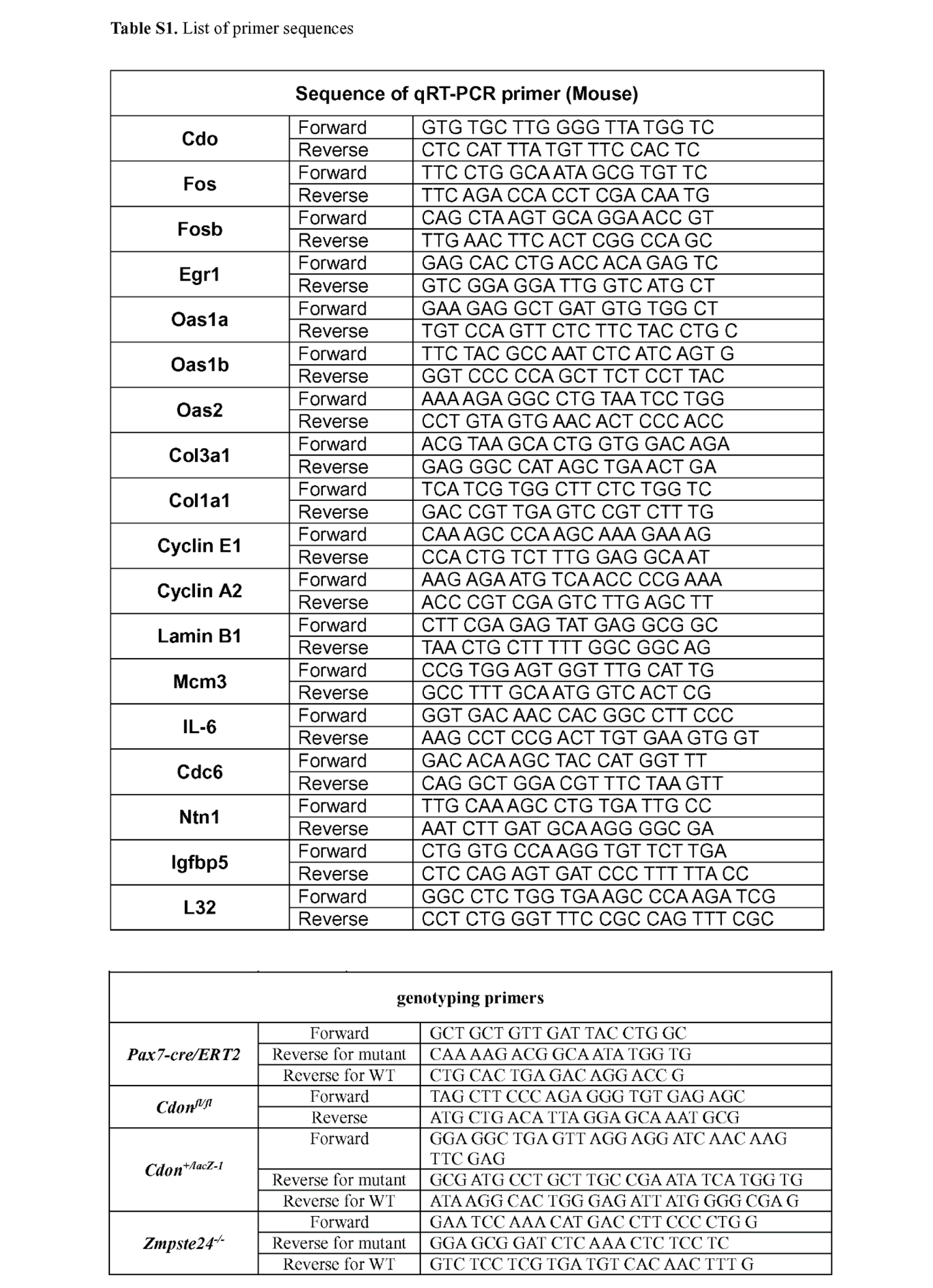

Supplement: Supplementary file 14 — Table S1. List of primer sequences [file JCSM-11-1089-s006.tif]

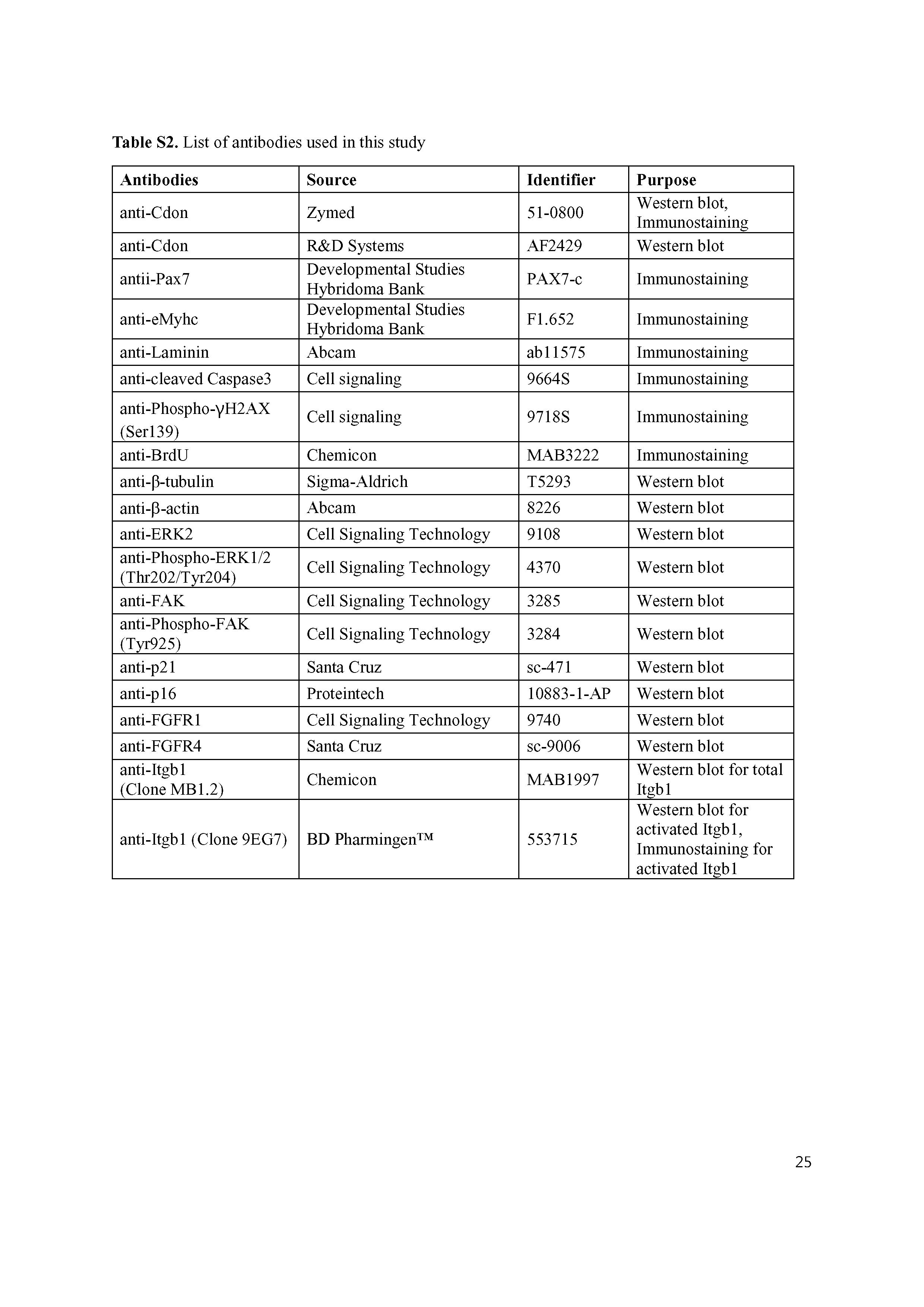

Supplement: Supplementary file 15 — Table S2. List of antibodies used in this body [file JCSM-11-1089-s007.tif]
