# Supplementary figures and images for: Corrigendum to Bae et al. J Cachexia Sarcopenia Muscle 11, 1089–1103, 2020. doi: 10.1002/jcsm.12563
Source: J Cachexia Sarcopenia Muscle. 2020 Oct 15;11(5):1381. doi: 10.1002/jcsm.12628 (PMC7567145; doi:10.1002/jcsm.12628)

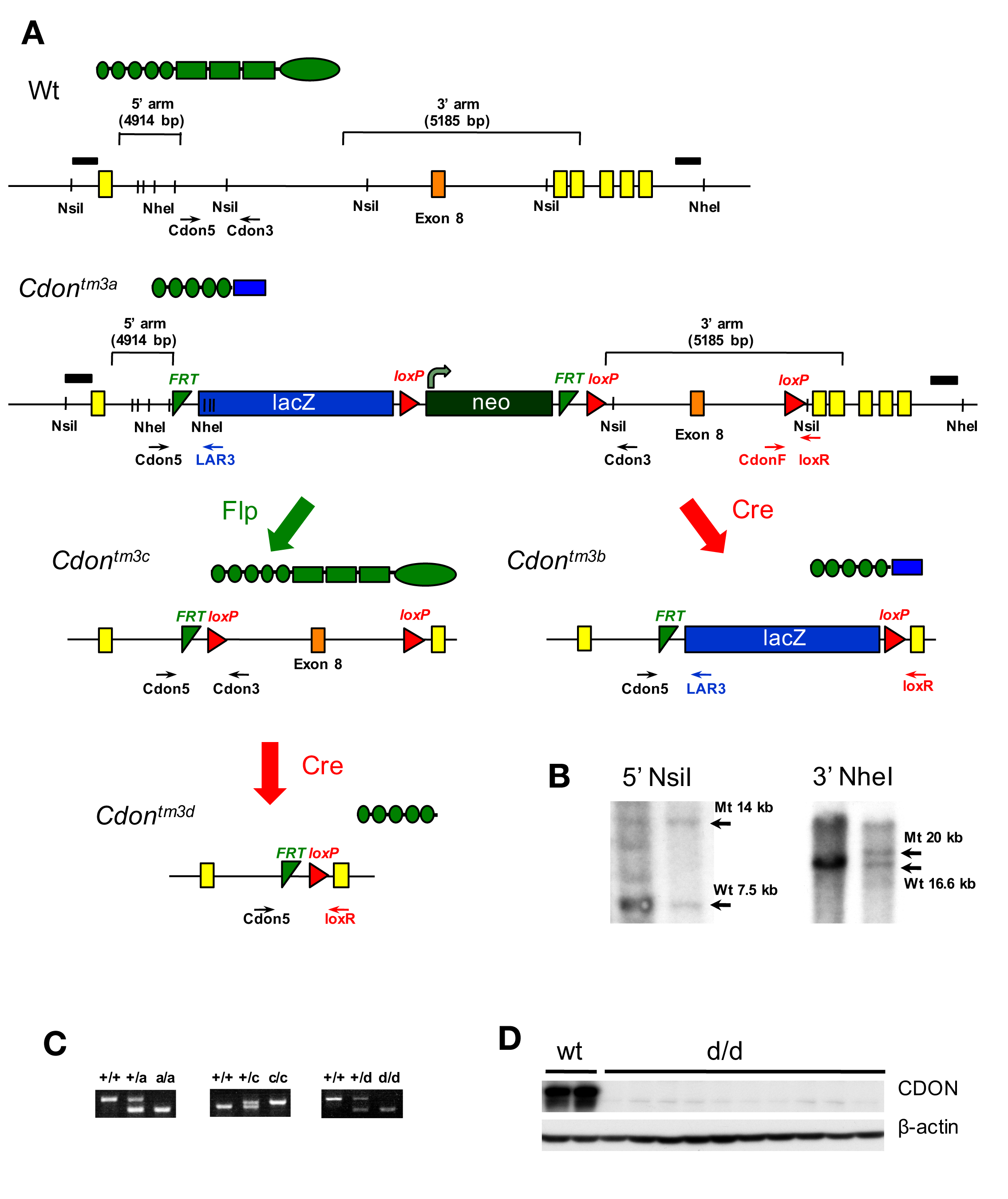

Supplement: Supplementary file 1 — Figure S1. Supporting Information [file JCSM-11-1381-s001.tif]
